# Forty New Faunistic Records of Spiders (Araneae: Araneomorphae) for the Republic of Kosovo

**DOI:** 10.3390/ani16172748

**Published:** 2026-09-02

**Authors:** Donard Geci, Tim Hoerrmann, Halil Ibrahimi, Astrit Bilalli, Milaim Musliu, Linda Grapci-Kotori, Danniella Sherwood

**Affiliations:** 1 Department of Biology, Faculty of Mathematics and Natural Sciences, University of Prishtina, Mother Teresa Street p.n., 10000 Prishtina, Kosovo; donard.geci@uni-pr.edu (D.G.); halil.ibrahimi@uni-pr.edu (H.I.); linda.grapci@uni-pr.edu (L.G.-K.); 2 Department of Biology, University of Konstanz, Universität sstr. 10, 78457 Konstanz, Germany; tim.hoerrmann@uni-konstanz.de; 3 Faculty of Agribusiness, University of Peja “Haxhi Zeka”, Street “UÇK”, 30000 Peja, Kosovo; astrit.bilalli@unhz.eu (A.B.); milaim.musliu@unhz.eu (M.M.)

**Keywords:** arachnofauna, Balkan Peninsula, biodiversity, faunistic records, species distribution, taxonomy

## Abstract

This study presents new faunistic records of spiders (Araneae) from Kosovo. A total of 40 species are reported for the first time from the country, simultaneously being the first country records for 14 genera, two of which also being newly recorded families. All newly recorded taxa are documented with photographs of the genital organs to facilitate identification and verification. These findings significantly increase knowledge of the distribution and diversity of spiders in Kosovo, highlighting how unexplored the arachnofauna of Kosovo is in comparison to neighbouring countries in the Balkans. Our results emphasise the importance of continued faunistic surveys and taxonomic studies in the region in order to improve our understanding of species diversity and biogeographic patterns.

## Simple Summary

The Republic of Kosovo is a landlocked country in the Balkan Peninsula, characterised by large mountainous borders and divided by three distinct ecoregions. As with many aspects of its invertebrate biology, Kosovo is a hitherto poorly-studied country for arachnids, especially spiders (Araneae). Consequently, several years of fieldwork by the authors has aimed to sample spiders from as many localities, habitats and microhabitat types as possible. Voucher specimens were taken for all morphospecies and compared against the published literature and online resources such as the World Spider Catalog and Araneae: Spiders of Europe. Consequently, this work reports 40 species of spider newly recorded from within the borders of the Republic of Kosovo, significantly expanding knowledge of the faunistic diversity and distribution of spiders within the country. The habitus and genitalia of all species are illustrated to justify the identifications made and to provide reference images for other workers in the Balkans to aid in their identification of faunistically widespread species.

## 1. Introduction

Spiders (Araneae Clerck, 1757) represent one of the most diverse groups of terrestrial arthropods and play an essential ecological role as generalist predators in nearly all terrestrial ecosystems. Currently, more than 54,000 spider species have been described worldwide [1], making spiders one of the largest and most diverse orders within Arthropoda. Despite their ecological importance and remarkable diversity, the spider fauna of many regions of the Balkan Peninsula remains insufficiently explored, and numerous taxa are still undocumented or poorly known.

The Balkan Peninsula is recognised as one of the principal biodiversity hotspots in Europe due to its complex geological history, pronounced habitat heterogeneity, and diverse climatic conditions. These factors have promoted high levels of endemism and species richness across numerous taxonomic groups, including arachnids. Nevertheless, arachnological research in several Balkan countries has historically been uneven [2,3], resulting in considerable gaps in the understanding of species distributions and regional diversity patterns. Kosovo, in particular, remains among the least investigated areas in southeastern Europe regarding spider diversity [2,4].

In recent years, several studies have gradually improved the knowledge of the spider fauna of Kosovo, documenting both endemic and biogeographically important taxa. Among these are the recently described endemic species *Brachythele kosovarica* Geci & Sherwood 2025; *Eratigena mirusha* Geci, Naumova, Ibrahimi 2025; *Harpactea dardanica* Geci & Zamani, 2025; and *Nemesia dukagjinica* Geci & Sherwood 2025 [5,6,7]. These discoveries and others [8] have highlighted the importance of documenting the arachnological diversity of Kosovo. However, while the aforementioned endemics have clearly defined distribution ranges, currently available data on other historically recorded and widespread Balkans spider species in Kosovo remains fragmentary and is based primarily on limited faunistic surveys.

In this work, we report forty new records of spider species from the Republic of Kosovo, raising the number of species from 282 [3,4,5,6,7,8,9] to 322. These findings further refine the current understanding of exact spider distributions in the Balkans and emphasise the importance of continued arachnological exploration in Kosovo and surrounding regions.

## 2. Materials and Methods

Spiders were collected from different localities across Kosovo using a variety of sampling methods, including hand collecting, pitfall traps, sieving, sweep netting, beating of tree branches, and an aspirator. Collector abbreviations: A.B. = Astrit Bilalli, D.G. = Donard Geci, D.S. = Danniella Sherwood; H.I. Halil Ibrahimi, L.G.K. = Linda Grapci Kotori, M.M. Milaim Musliu, T.H = Tim Hoerrman. Specimens were preserved in 70% ethanol for long-term storage and examined by D.G, D.S., and T.H. using an Olympus SZ15 stereomicroscope (Olympus, Hamburg, Germany). Photographs of specimens were made by D.G. and D.S. using an Olympus SC50 camera attached to the aforementioned microscope. Drawings were made by H.I. using the same microscope and Adobe Illustrator (v2023). Species identification was primarily based on the online identification platform, Araneae: Spiders of Europe [9], in combination with specimens being identified against all sources cited in the respective synonymy lists. Distribution ranges were taken verbatim from [1] and only modified if further clarity was needed. For the examination of female copulatory organs, which are critical for species identification, epigynes were dissected and cleared in a 10% KOH solution to dissolve soft tissues and enhance the visibility of internal structures. Nomenclature follows the World Spider Catalog [1]; synonymy lists contain one modern reference of comparative genitalia of the species from material from other countries (for reader comparison), and taxa are presented in alphabetical order. The prior number of species for Kosovo (282), together with knowledge of the prior-recorded genera and families, is derived from [9], where we direct readers for a summary of the source for each species previously reported. All specimens examined were deposited in the collection of the Department of Biology, Faculty of Mathematics and Natural Sciences, University of Prishtina, Prishtina, Kosovo (UP). ZooBank registration: 

urn:lsid:zoobank.org:pub:62B0701B-457D-4692-81CE-34523348DD2D.

## 3. Results

**Family Agelenidae C. L. Koch, 1837**

**Figure 1:**
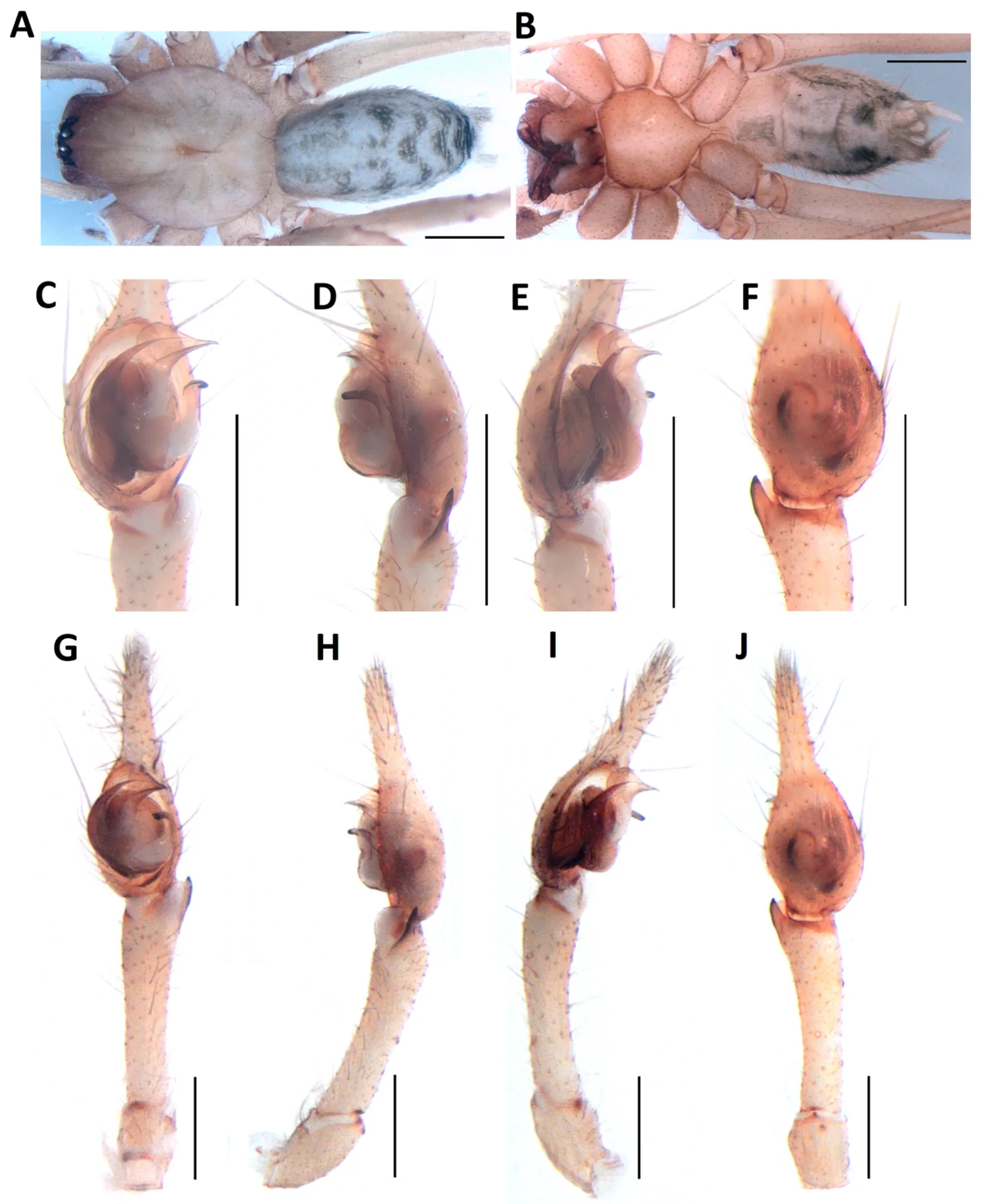
*Tegenaria faniapollinis* Brignoli, 1978, male from Prizren, Kosovo: (**A**) habitus, dorsal view; (**B**) idem, ventral view; (**C**) close-up of palpal sclerites, ventral view; (**D**) idem, retrolateral view; (**E**) idem, prolateral view; (**F**) idem, dorsal view; (**G**) palp from patella to cymbium, ventral view; (**H**) idem, retrolateral view; (**I**) idem, prolateral view; (**J**) idem, dorsal view. Scale bars: (**A**,**B**) = 1 mm, (**C**–**J**) = 0.5 mm.

***Tegenaria faniapollinis* Brignoli, 1978 (Figure 1)**

*Tegenaria faniapollinis*: Lecigne, 2021: 23, figs. 23a–e (m) [10].

**Material examined.** 1 ♂ (UP Ar1015), Korishë, Prizren, 42.26249° N, 20.78676° E, 483 m, 15 May 2023, near houses, collected by hand, leg: D.G.

**Distribution.** Italy (Sicily), North Macedonia, Bulgaria, Greece, Turkey [1].

**Remarks.** The morphology of the conductor, median apophysis, embolus and retrolateral apophysis are in agreement with this species.

**Family Araneidae Clerck, 1757**

**Figure 2:**
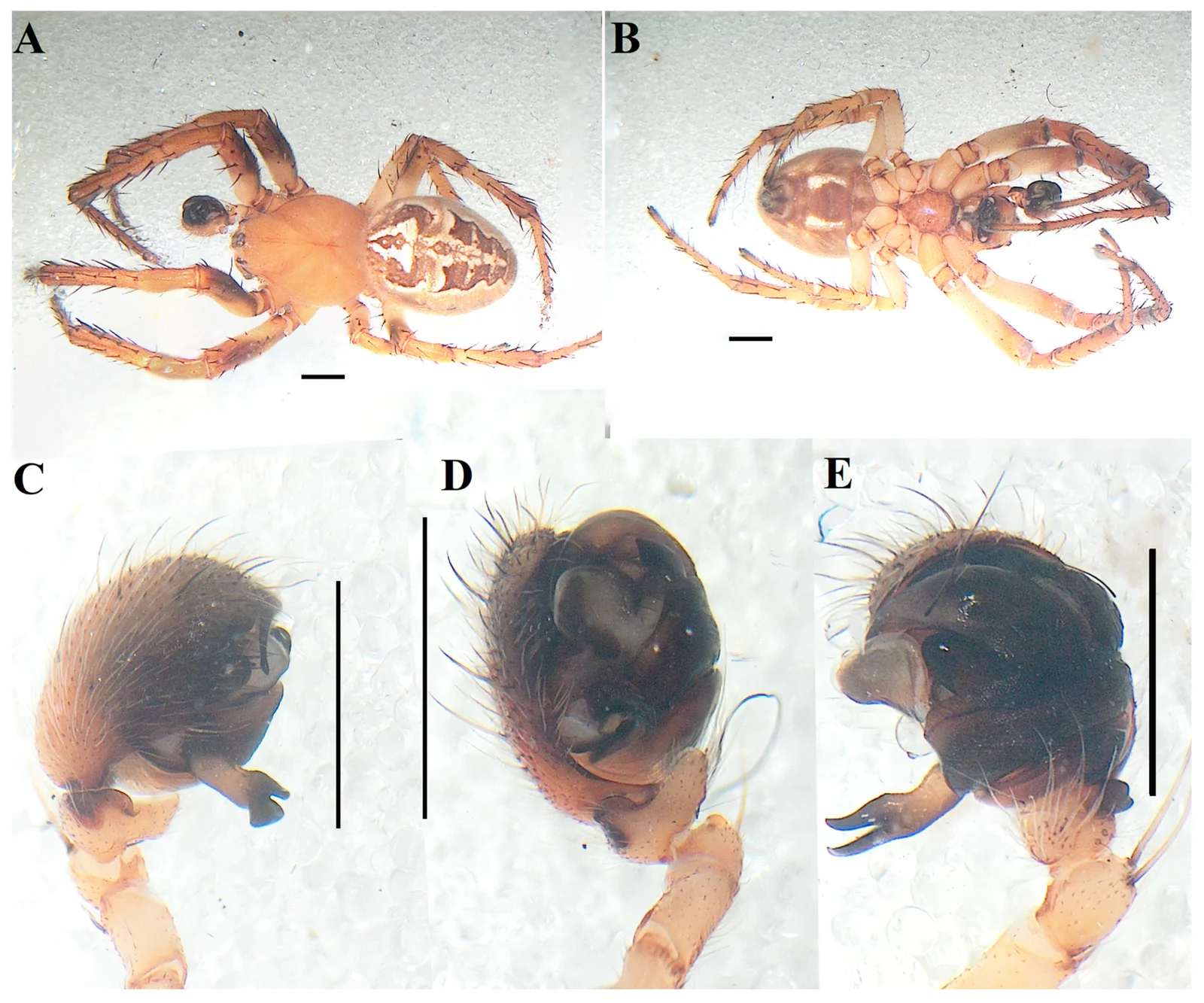
*Larinioides cornutus* (Clerck, 1757) male from Henc, Kosovo: (**A**) habitus, dorsal view; (**B**) idem, ventral view; (**C**) palp, retrolateral view; (**D**) idem, retroventral view; (**E**) idem, ventral view. Scale bars = 1 mm.

***Larinioides cornutus* (Clerck, 1757) (Figure 2)**

*Larinioides cornutus*: Marusik, 2015: 672, figs. 4G,H and 6A (mf) [11].

**Material examined.** 1 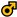 (UP Ar1016), Henc, Fushë Kosovë, 42.58227° N, 21.04886° E, 536 m, 24 August 2024, near water, collected by hand, leg: T.H.; 2 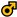 (UP Ar1017), Veriq, Istog, 42.75379° N, 20.55692° E, 510 m, near water, collected by hand, 15 July 2023, leg: D.G.

**Distribution.** North America, Europe, Northern Africa, Turkey, Israel, Caucasus, Russia (Europe to Far East), Iran, China, Korea, Japan [1].

**Remarks.** The morphology of the median apophysis and other sclerites, together with the habitus, allows the specimens to be readily identified as this easily recognised species.

**Figure 3:**
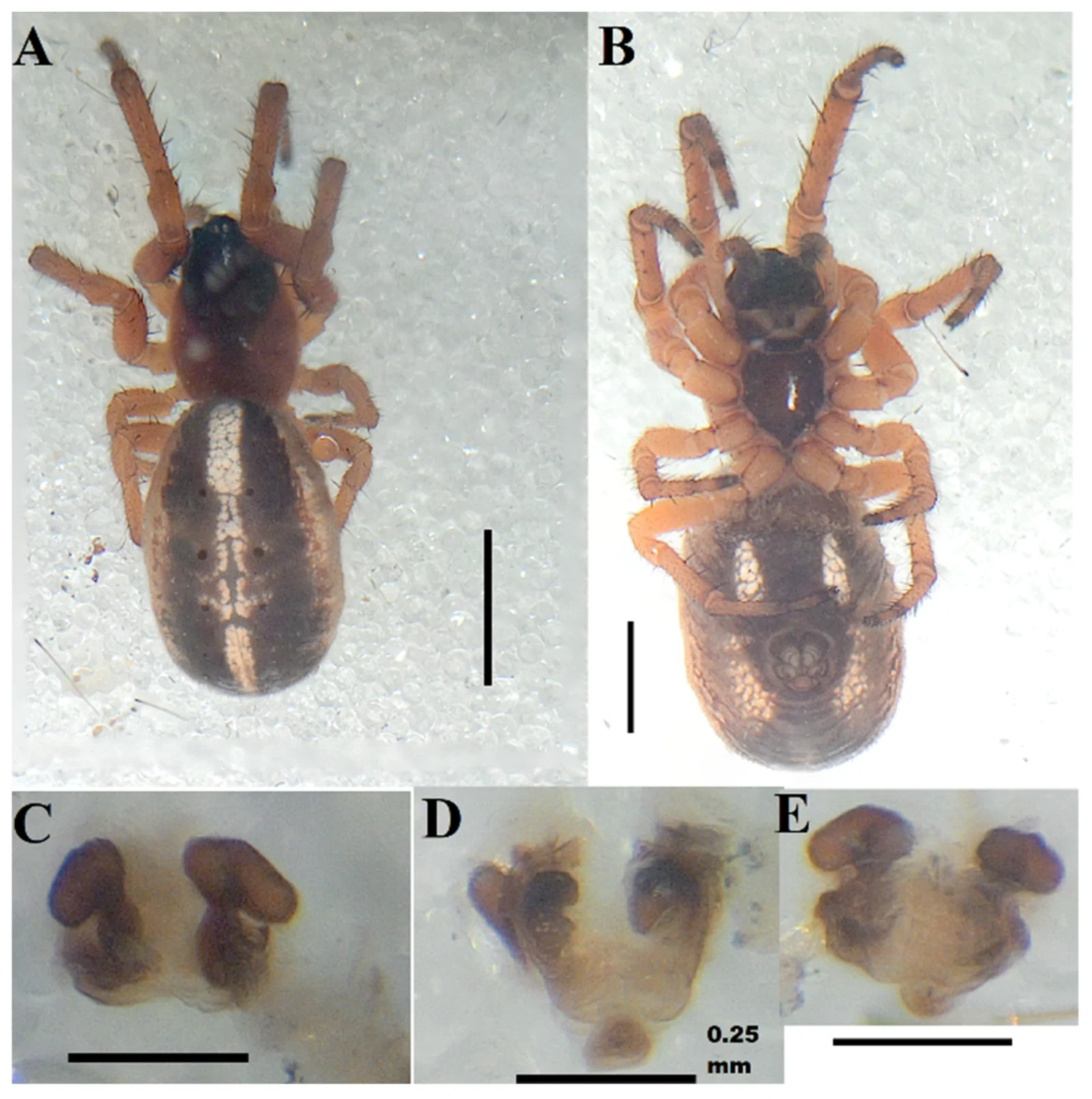
*Singa nitidula* C. L. Koch, 1844 female from Landovicë, Kosovo: (**A**) habitus, dorsal view; (**B**) idem, ventral view; (**C**) epigyne, dissected and cleared, ventral view; (**D**) vulva, cleared, dorsal view; (**E**) idem, dorso-posterior view. Scale bars: habitus (**A**,**B**) = 1 mm; epigyne = (**C**–**E**) = 0.25 mm.

***Singa nitidula* C. L. Koch, 1844 (Figure 3)**

*Singa nitidula*: Almquist, 2006: 177, figs. 182a–e (mf) [12].

**Material examined.** 1 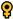 (UP Ar1018), Landovicë, Prizren, 42.27816° N, 20.66583° E, 357 m, grassland, collected with aspirator, 25 August 2024, leg: T. H.

**Distribution.** Europe, Turkey, Russia (Europe to Far East), Caucasus to Central Asia [1].

**Remarks.** The morphology of the epigyne and vulva, together with the abdomen pattern, allows recognition of the female.

**Family Dictynidae O. Pickard-Cambridge, 1871**

**Figure 4:**
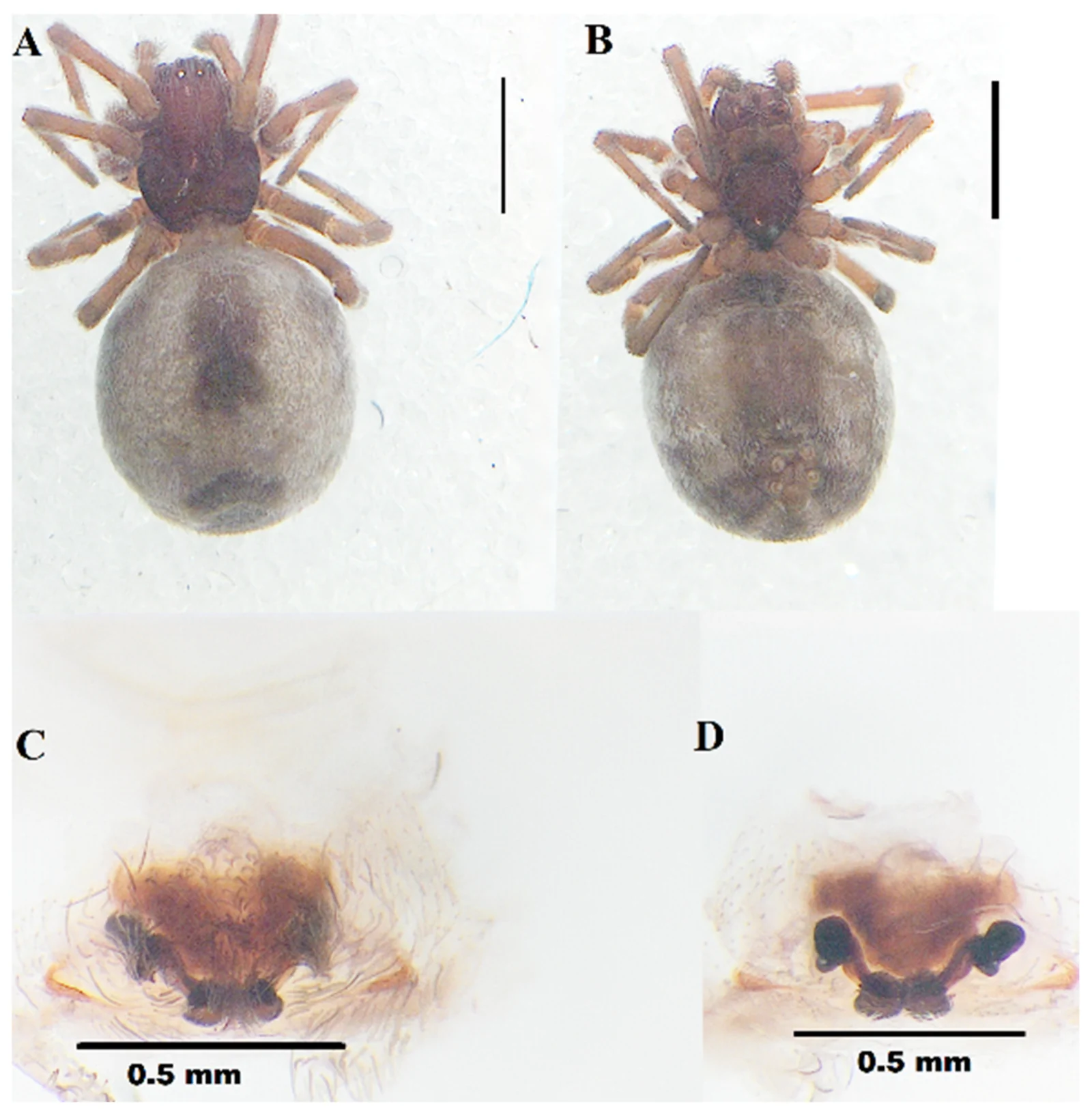
*Dictyna arundinacea* (Linnaeus, 1758) female from Henc, Kosovo: (**A**) habitus, dorsal view; (**B**) idem, ventral view; (**C**) epigyne, dissected and cleared, ventral view; (**D**) vulva, cleared, dorsal view. Scale bars: habitus (**A**,**B**) = 1 mm; epigyne = (**C**,**D**) = 0.5 mm.

***Dictyna arundinacea* (Linnaeus, 1758) (Figure 4)**

*Dictyna arundinacea* Esyunin, 2017: 268, fig. 4 (f) [13].

**Material examined.** 2 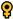

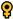
 (UP Ar1019), Henc, Fushë Kosovë, 42.58227° N, 21.04886° E, 536 m, grassland, collected by hand, 30 August 2024, leg: T. H.

**Distribution.** North America, Europe, Turkey, Caucasus, Russia (Europe to Far East), Iran, Central Asia, China, Korea, Japan [1].

**Remarks.** The morphology of the spermathecae and their spacing allow this material to be distinguished from other nearby congeners.

**Family Dysderidae C. L. Koch, 1837**

**Figure 5:**
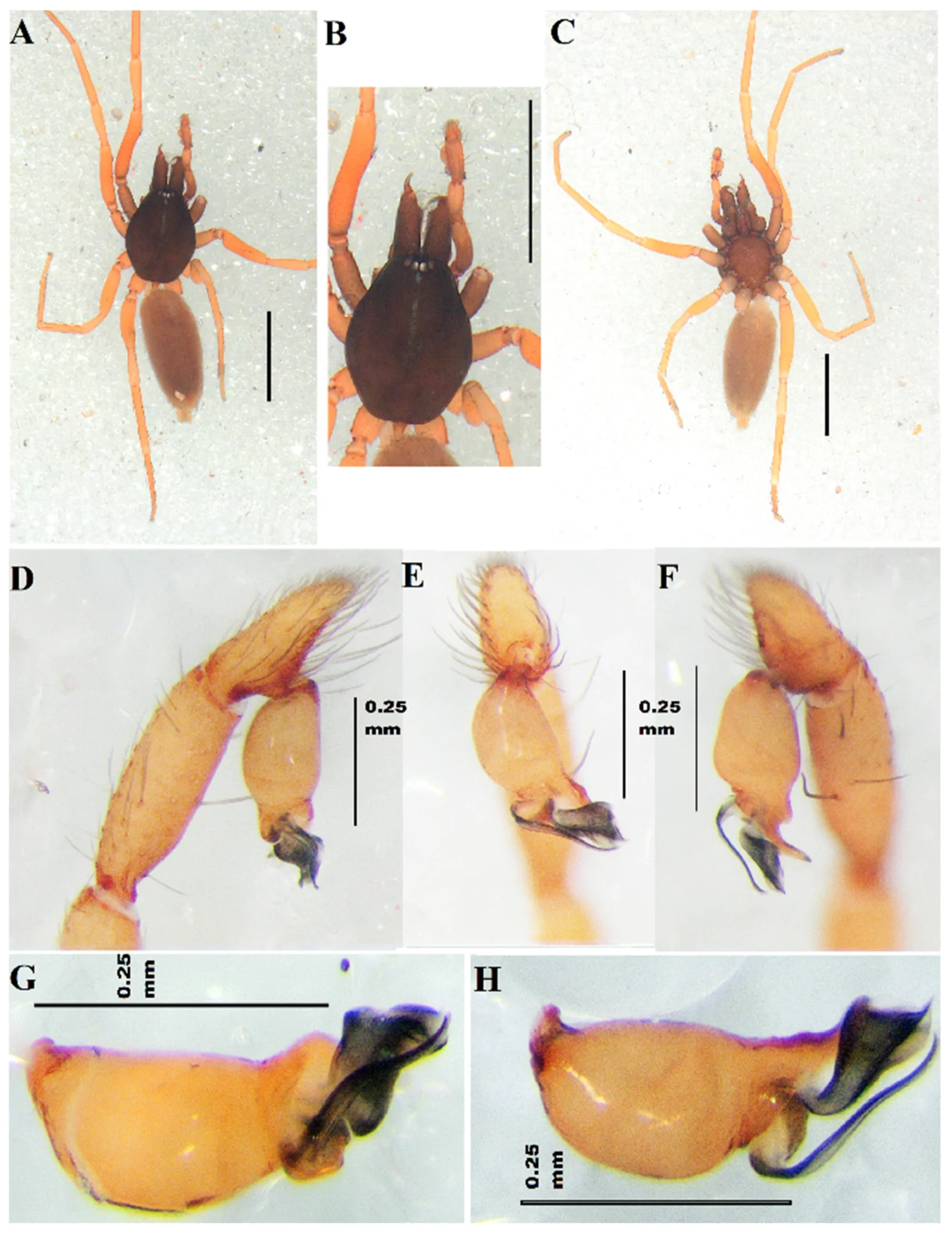
*Harpactea* cf. *coccifera* Brignoli, 1984 male from Debëlldëh, Kosovo: (**A**) habitus, dorsal view; (**B**) close-up of cephalothorax, dorsal view; (**C**) habitus, ventral view; (**D**) palp, prolateral view; (**E**) idem, ventral view; (**F**) idem, retrolateral view; (**G**) bulb, dissected, dorsal view; (**H**) idem, ventral view. Scale bars: habitus (**A**–**C**) = 1 mm; palp = (**D**–**H**) = 0.25 mm.

***Harpactea* cf. *coccifera* Brignoli, 1984 (Figure 5)**

*Harpactea coccifera*: Brignoli, 1984: 283, figs. 1–3 (m) [14].

**Material examined.** 1 ♂ (UP Ar1020), Debëlldëh, Viti, (site 1) 42.25405° N, 21.40004° E, 967 m, deciduous forest, collected by sieving leaflitter, 25 April 2026, leg: A.B., D.G., M.M.

**Distribution.** North Macedonia, Greece (Crete) [1].

**Remarks.** The genus *Harpactea* requires a thorough modern revision, which is outside the scope of this work. We identified this male as *H.* cf. *coccifera* based on resemblance of the shape of the embolus, apophyses, and the tegulum with the original description of Brignoli. We prefer a conservative approach, recognising that only a single male was available for the current study. Further fieldwork in this region by our team is planned for the future.

**Family Cicurinidae F. O. Pickard-Cambridge, 1893**

**Figure 6:**
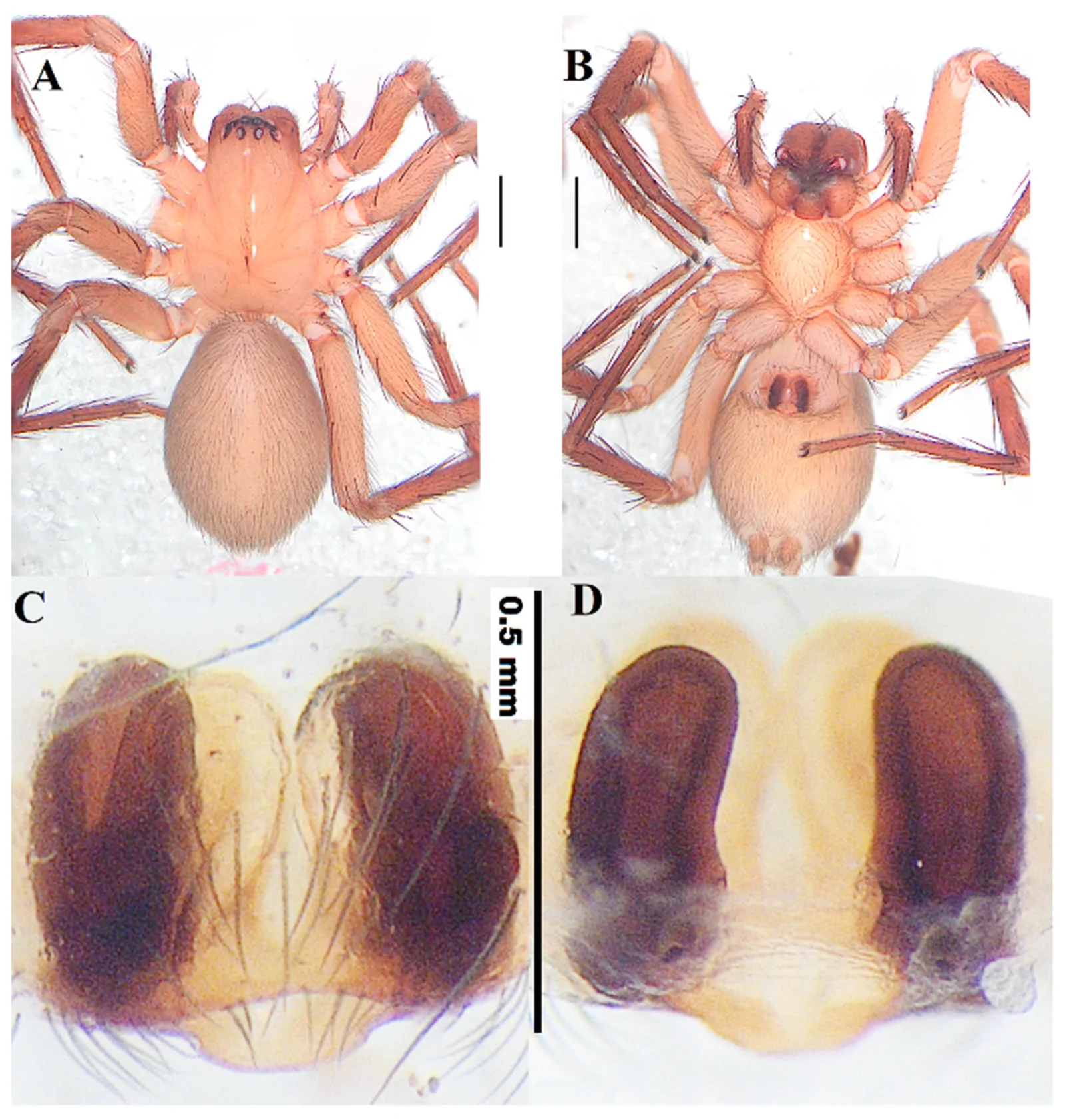
*Cicurina cicur* (Fabricius, 1792) female from Gurrat e bardha, Kosovo: (**A**) habitus, dorsal view; (**B**) idem, ventral view; (**C**) epigyne, dissected and cleared, ventral view; (**D**) vulva, cleared, dorsal view. Scale bars: habitus (**A**,**B**) = 1 mm; epigyne (**C**,**D**) = 0.5 mm.

***Cicurina cicur* (Fabricius, 1792) (Figure 6)**

*Cicurina cicur*: Almquist, 2007: 306, figs. 269a–g (mf) [15].

**Material examined.** 1 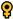 (UP Ar1021), Gurrat e bardha, Mokën, Istog, 42.81326° N, 20.45472° E, 1412 m, beech forest, collected by sieving leaflitter, 2 November 2025, leg: A.B., D.G., L.G.K., H.I.; 1 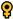 (UP Ar1022), Bajgorë, Mitrovicë, 42.96002° N, 21.01451° E, 1252 m, beech forest, collected by sieving leaflitter, 10 May 2026, leg: D.G.

**Distribution.** Europe to Central Asia [1].

**Remarks.** First record of the family and genus for the country. The shape of the scape and the spermathecal receptacles allow its identification.

**Family Cybaeidae Banks, 1892**

**Figure 7:**
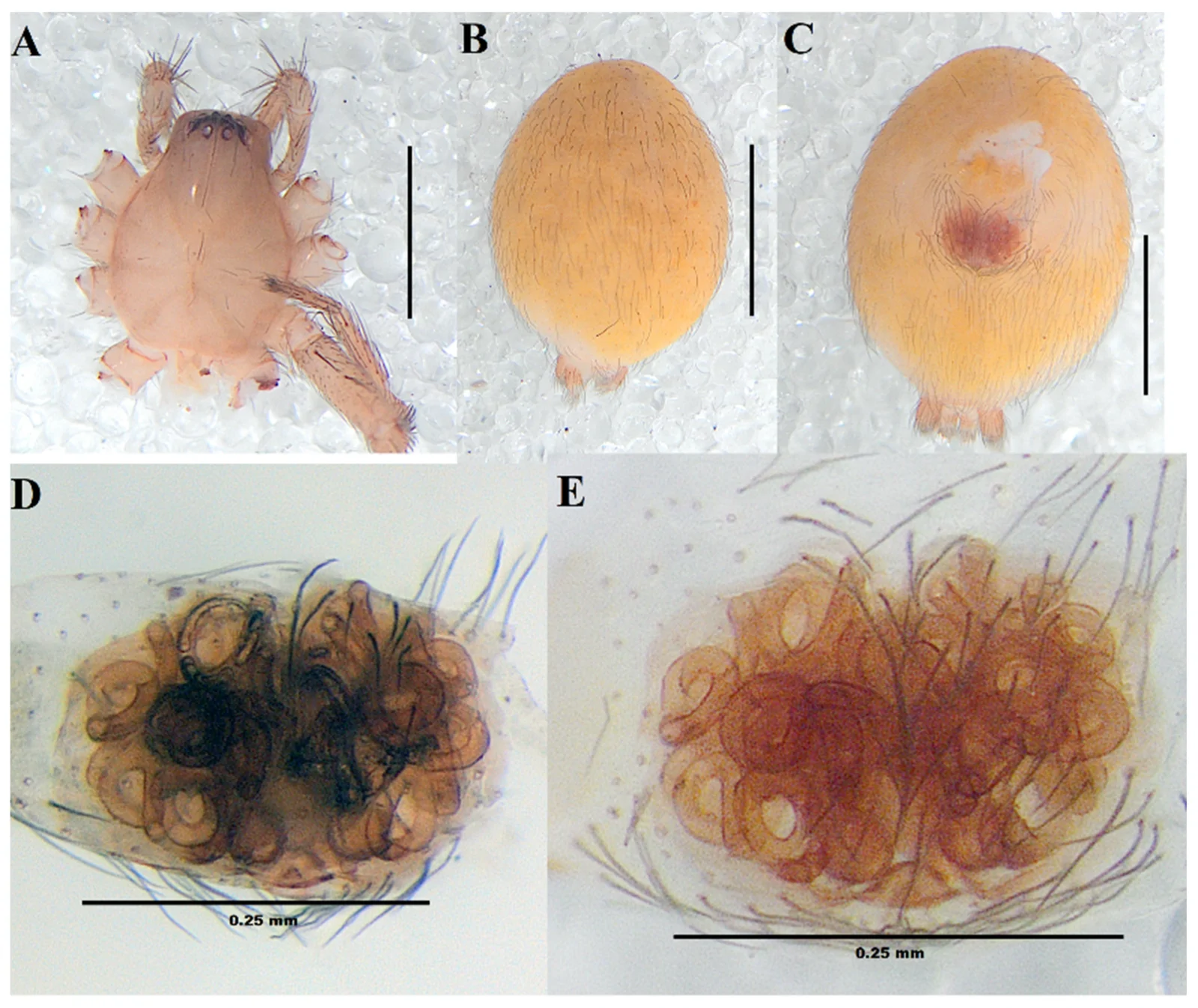
*Mastigusa arietina* (Thorell, 1871) female from Lugu i Butë, Kosovo: (**A**) cephalothorax, dorsal view; (**B**) abdomen, dorsal view; (**C**) idem, ventral view; (**D**) epigyne, dissected and cleared, ventral view; (**E**) vulva, cleared, dorsal view. Scale bars: habitus (**A**–**C**) = 1 mm; epigyne = (**D**,**E**) = 0.25 mm.

***Mastigusa arietina* (Thorell, 1871) (Figure 7)**

*Mastigusa arietina*: Indzhov et al., 2025: 581, figs. 1, 2, 4–7, 10, 11, and 13–18 (mf) [16].

**Material examined.** 1 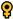 (UP Ar1023), Lugu i Butë, Mokën, 42.82294° N, 20.40783° E, 1757 m, alpine forest, under rock, collected with aspirator, 2 November 2025, leg: A.B., D.G., L.G.K., H.I.

**Distribution.** Europe, Turkey, Russia (Europe, Caucasus, South Siberia), Georgia, Iran [1].

**Remarks.** First record of the genus for the country. As per [16], this species can be distinguished from *M. macrophthalma* (Kulczyński, 1897) by the wider and less dense copulatory ducts.

**Family Gnaphosidae Banks, 1892**

**Figure 8:**
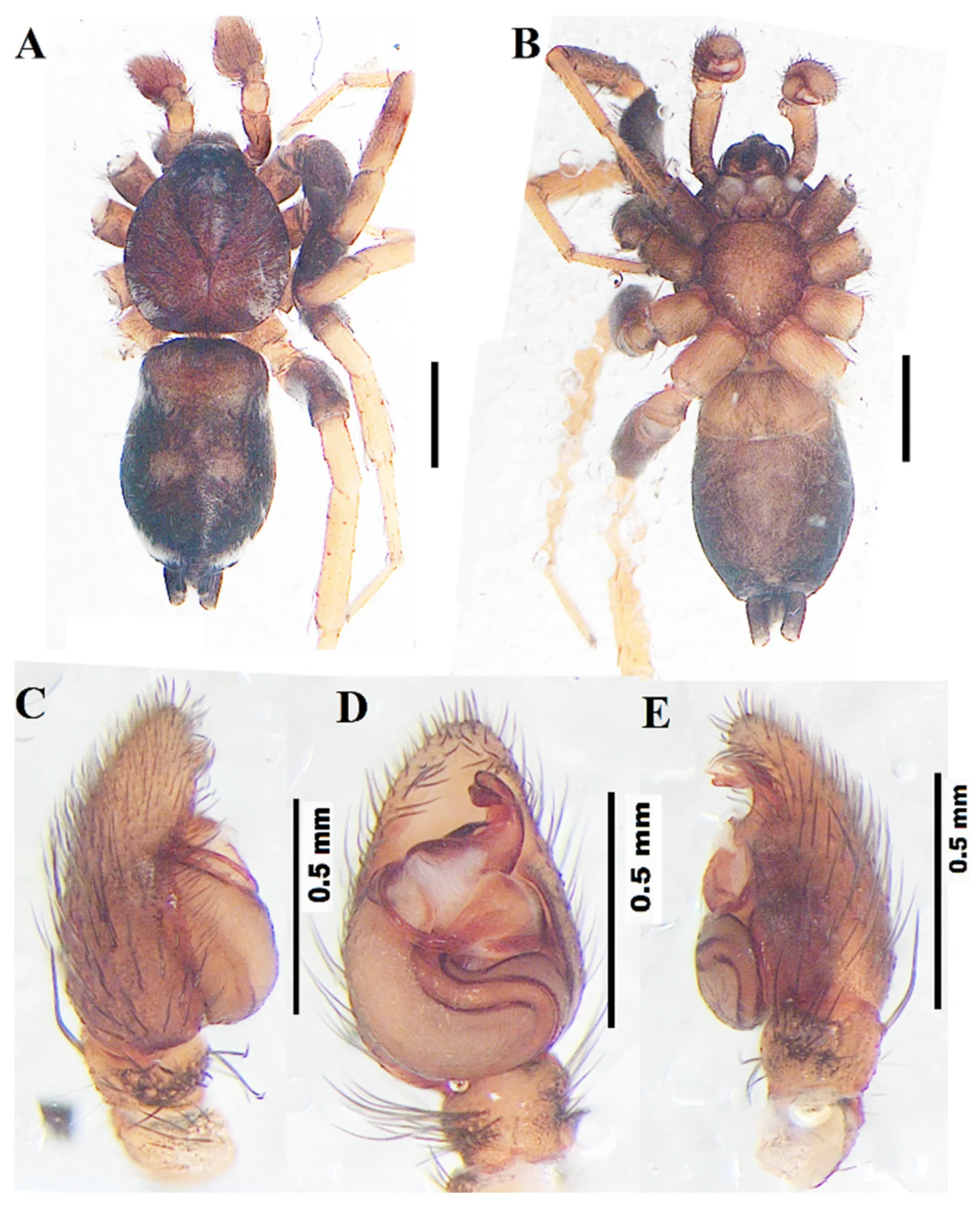
*Callilepis schuszteri* (Herman, 1879) male from Pustenik, Kosovo: (**A**) habitus, dorsal view; (**B**) idem, ventral view; (**C**) palp, prolateral view; (**D**) idem, ventral view; (**E**) idem, retrolateral view. Scale bars: habitus (**A**,**B**) = 1 mm; palp = (**C**–**E**) = 0.5 mm.

***Callilepis schuszteri* (Herman, 1879) (Figure 8)**

*Callilepis schuszteri*: Ponomarev & Shmatko, 2022: 288, figs. 5–7 (mf) [17].

**Material examined.** 1 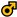 (UP Ar1024), Pustenik, Hani i Elezit, 42.16520° N, 21.25770° E, 687 m, meadow, under rock, collected with aspirator, 10 May 2025, leg: D.G.

**Distribution.** Europe, Turkey, Caucasus, Russia (Europe to Far East), China, Korea, Japan [1].

**Remarks.** The morphology of palpal sclerites in combination with colouration allow us to rule out other congeners.

**Figure 9:**
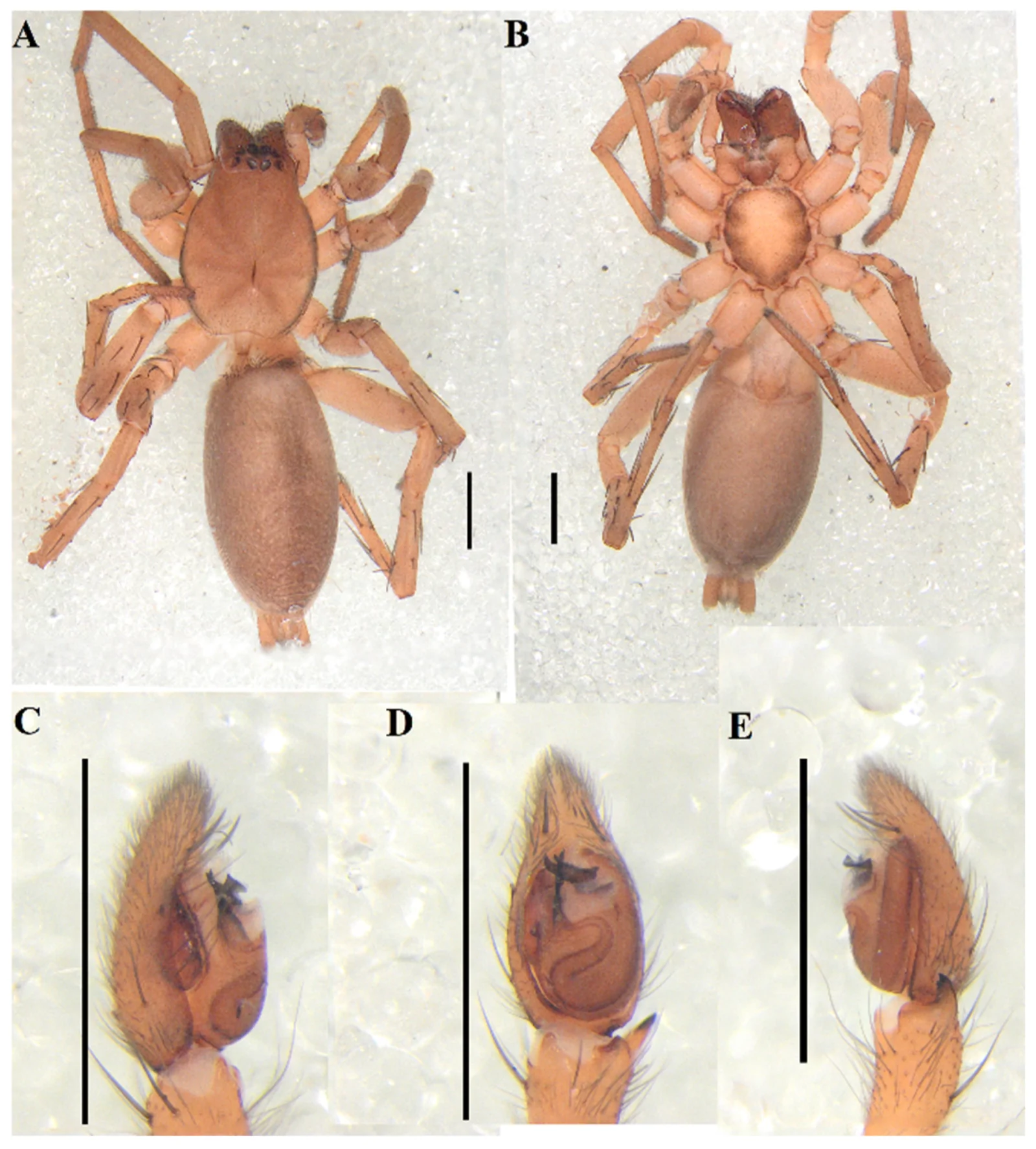
*Drassodes pubescens* (Thorell, 1856) male from Restelicë, Kosovo: (**A**) habitus, dorsal view; (**B**) idem, ventral view; (**C**) palp, prolateral view; (**D**) idem, ventral view; (**E**) idem, retrolateral view. Scale bars: 1 mm.

***Drassodes pubescens* (Thorell, 1856) (Figure 9)**

*Drassodes pubescens*: Barrientos et al., 2017: 113, figs. 5(A3,B3) (mf) [18].

**Material examined.** 2 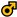

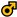
 (UP Ar1025), Restelicë, Sharr Mountain, Prizren, (site 1) 41.95681° N, 20.65770° E, 1594 m, valley, under rock, collected by hand, 31 May 2025, leg: A.B., D.G., D.S.

**Distribution.** Europe, Turkey, Israel, Caucasus, Russia (Europe to Far East), Iran, Central Asia, China, Japan [1].

**Remarks.** The distinctive position and shape of the embolus and median apophysis allow its recognition.

**Figure 10:**
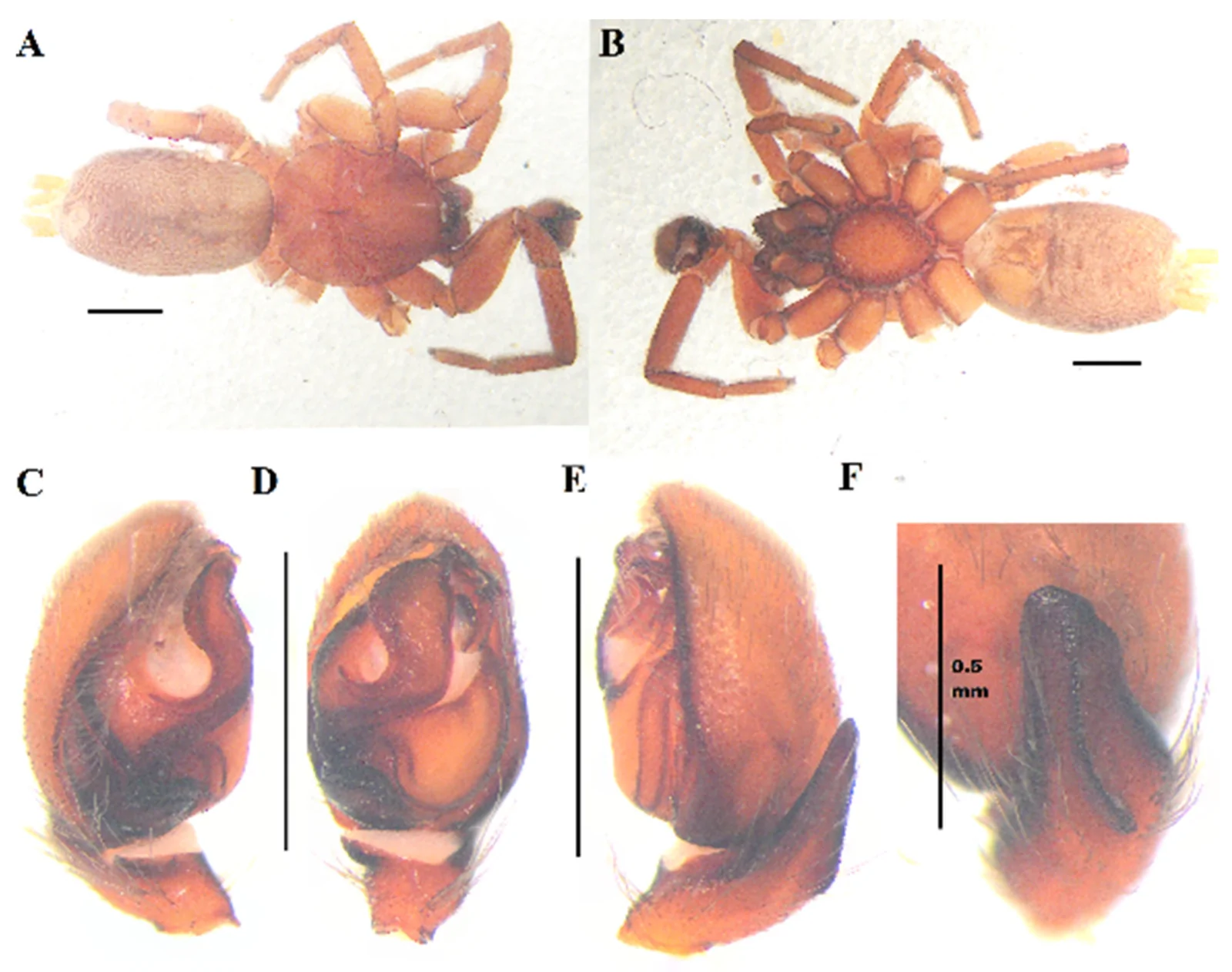
*Haplodrassus silvestris* (Blackwall, 1833) male from Dermjak, Kosovo: (**A**) habitus, dorsal view; (**B**) idem, ventral view; (**C**) palp, prolateral view; (**D**) idem, ventral view; (**E**) idem, retrolateral view; (**F**) close-up of retrolateral tibial apophysis. Scale bars: habitus (**A**,**B**) = 1 mm; palp: (**C**,**D,E**) = 1 mm; (**F**) RTA = 0.5 mm.

***Haplodrassus 1833.* (Figure 10).**

*Haplodrassus silvestris*: Almquist, 2007: 411, figs. 355a–f (mf) [15].

**Material examined.** 3 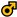 (UP Ar1026), Dermjak, Hani i Elezit, 42.18079° N, 21.31408° E, 729 m, forest, collected by hand, 18 August 2020, leg: A.B., D.G., M.M.

**Distribution.** Europe, Turkey, and the Caucasus [1].

**Remarks.** The morphology of the retrolateral tibial apophysis and the wide median apophysis allow its identification.

**Figure 11:**
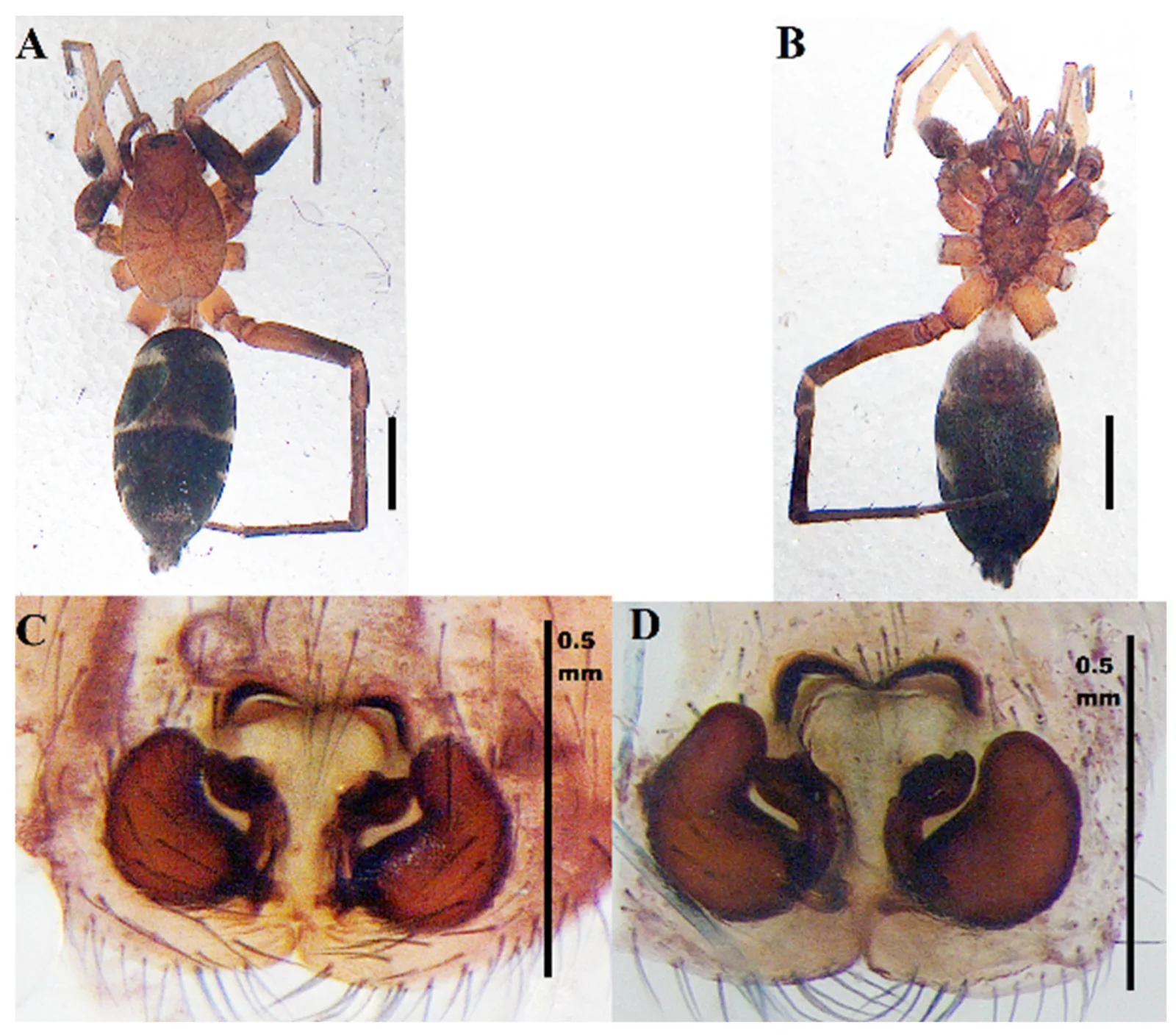
*Micaria albovittata* (Lucas, 1846) female from Krevenik, Kosovo: (**A**) habitus, dorsal view; (**B**) idem, ventral view; (**C**) epigyne, dissected and cleared, ventral view; (**D**) vulva, cleared, dorsal view. Scale bars: habitus (**A**,**B**) = 1 mm; epigyne = (**C**,**D**) = 0.5 mm.

***Micaria albovittata* (Lucas, 1846) (Figure 11)**

*Micaria albovittata*: Lecigne, 2016: 112, figs. 8J–M (mf) [19].

**Material examined.** 1 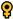 (UP Ar1027), Krevenik, Hani i Elezit, (site 1) 42.11153° N, 21.25359° E, 684 m, rocky escarpment, collected by hand, 30 May 2026 leg: D.G., D.S.

**Distribution.** Europe, Turkey, Caucasus, Russia (Europe to Central Asia), Iran, Turkmenistan [1].

**Remarks.** The morphology of the anterior hoods of the epigyne and the kidney-shaped spermathecae allow its identification.

**Figure 12:**
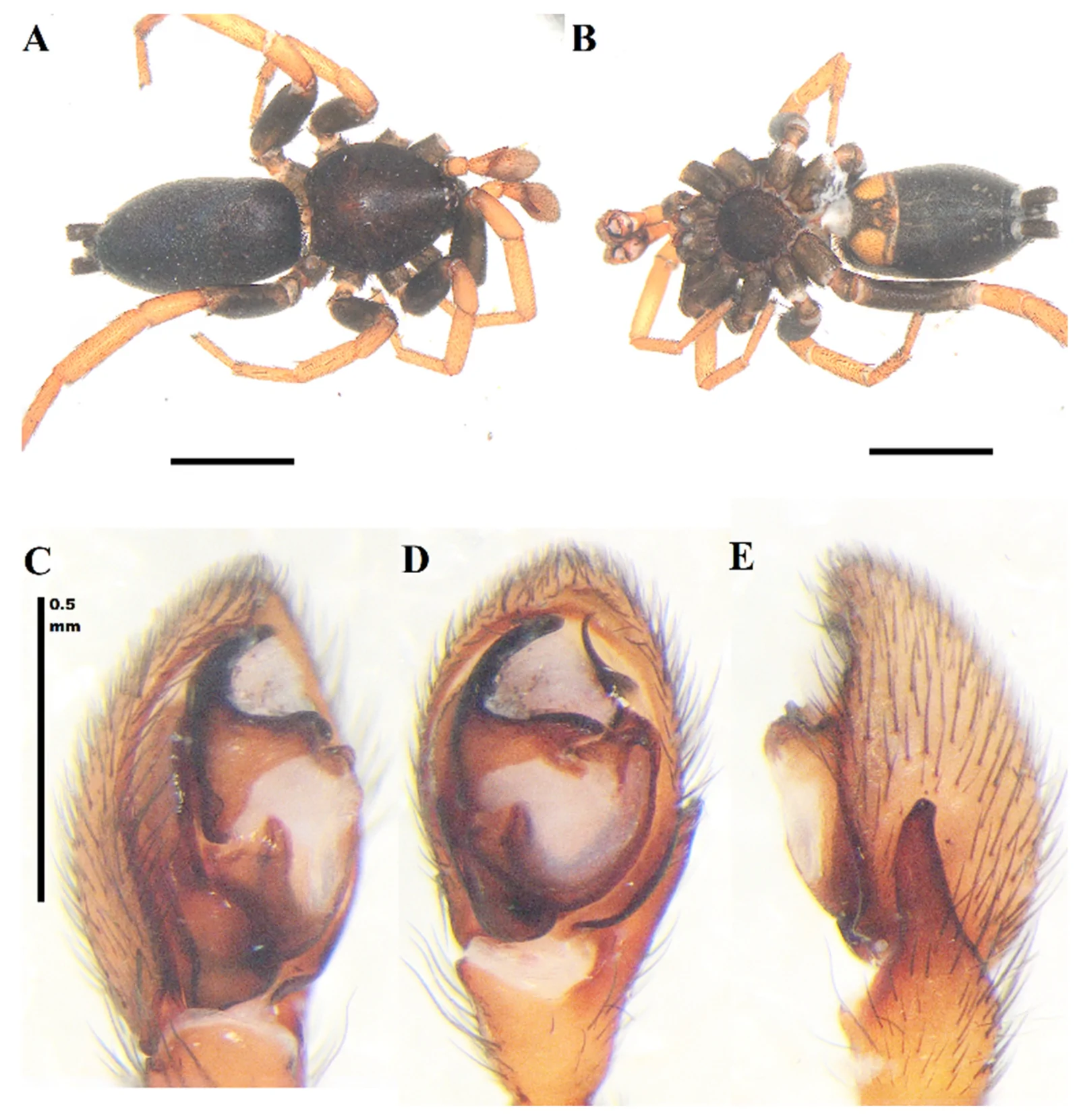
*Trachyzelotes pedestris* (C. L. Koch, 1837) male from Zhur, Kosovo: (**A**) habitus, dorsal view; (**B**) idem, ventral view; (**C**) palp, prolateral view; (**D**) idem, ventral view; (**E**) idem, retrolateral view. Scale bars: habitus (**A**,**B**) = 1 mm; palp (**C**–**E**) = 0.5 mm.

***Trachyzelotes pedestris* (C. L. Koch, 1837) (Figure 12)**

*Trachyzelotes pedestris*: Ponomarev & Shmatko, 2020: 127, figs. 1, 2, 21, 22, 31–34, 47, 51, and 54–56 (mf) [20].

**Material examined.** 1 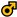 (UP Ar1028), Zhur, Prizren, 42.17043° N, 20.60477° E, 539 m, meadow, under rocks, collected by hand, 1 June 2025, leg: A.B., D.G., D.S.

**Distribution.** Europe, Caucasus, Turkey, Iran [1].

**Remarks.** First record of the genus for the country. This species is easily recognised by the morphology of the embolus, median apophysis, and the retrolateral tibial apophysis.

**Family Linyphiidae Blackwall, 1859**

**Figure 13:**
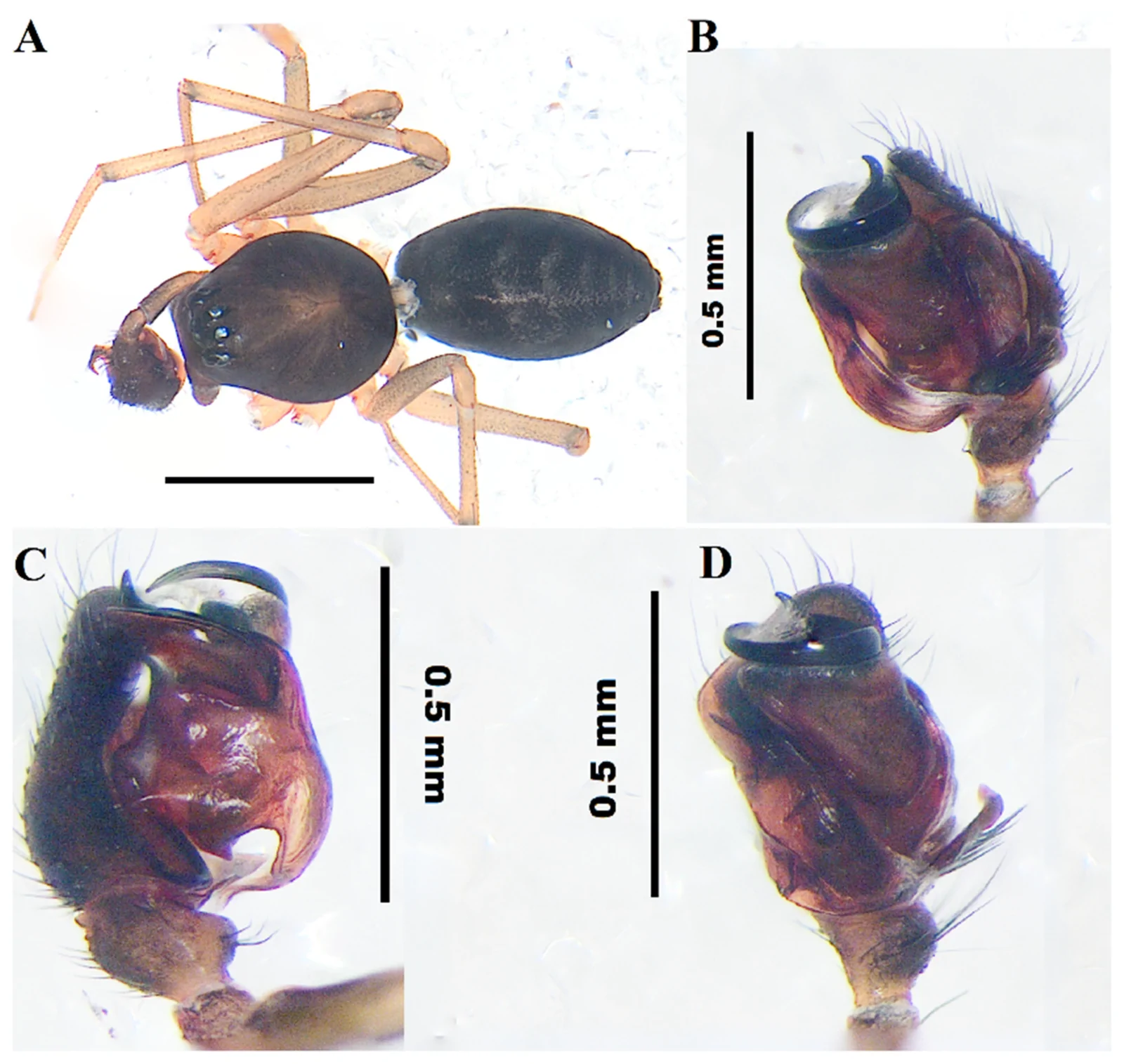
*Bathyphantes nigrinus* (Westring, 1851) male from Bjeshka e Uçës, Kosovo: (**A**) habitus, dorsal view; (**B**) palp, prolateral view; (**C**) idem, ventral view; (**D**) idem, retrolateral view. Scale bars: habitus (**A**) = 1 mm; palp (**B**–**D**) = 0.5 mm.

***Bathyphantes nigrinus* (Westring, 1851) (Figure 13)**

*Bathyphantes nigrinus*: Lecigne, 2020: 57, figs. 4C–D (mf) [21].

**Material examined.** 3 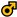

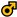
 (UP Ar1029), Bjeshka e Uçës, Mokën, 42.86110° N, 20.57547° E, 1314 m, alpine stream, under stones, collected by hand, 25 October 2025, leg: A.B., D.G., L.G.K, H.I.

**Distribution.** Europe (incl. Russian) to South Siberia [1].

**Remarks.** First record of the genus for the country. The distinctive shapes of the tegulum, apophysis, and tip of the embolus allow its identification.

**Figure 14:**
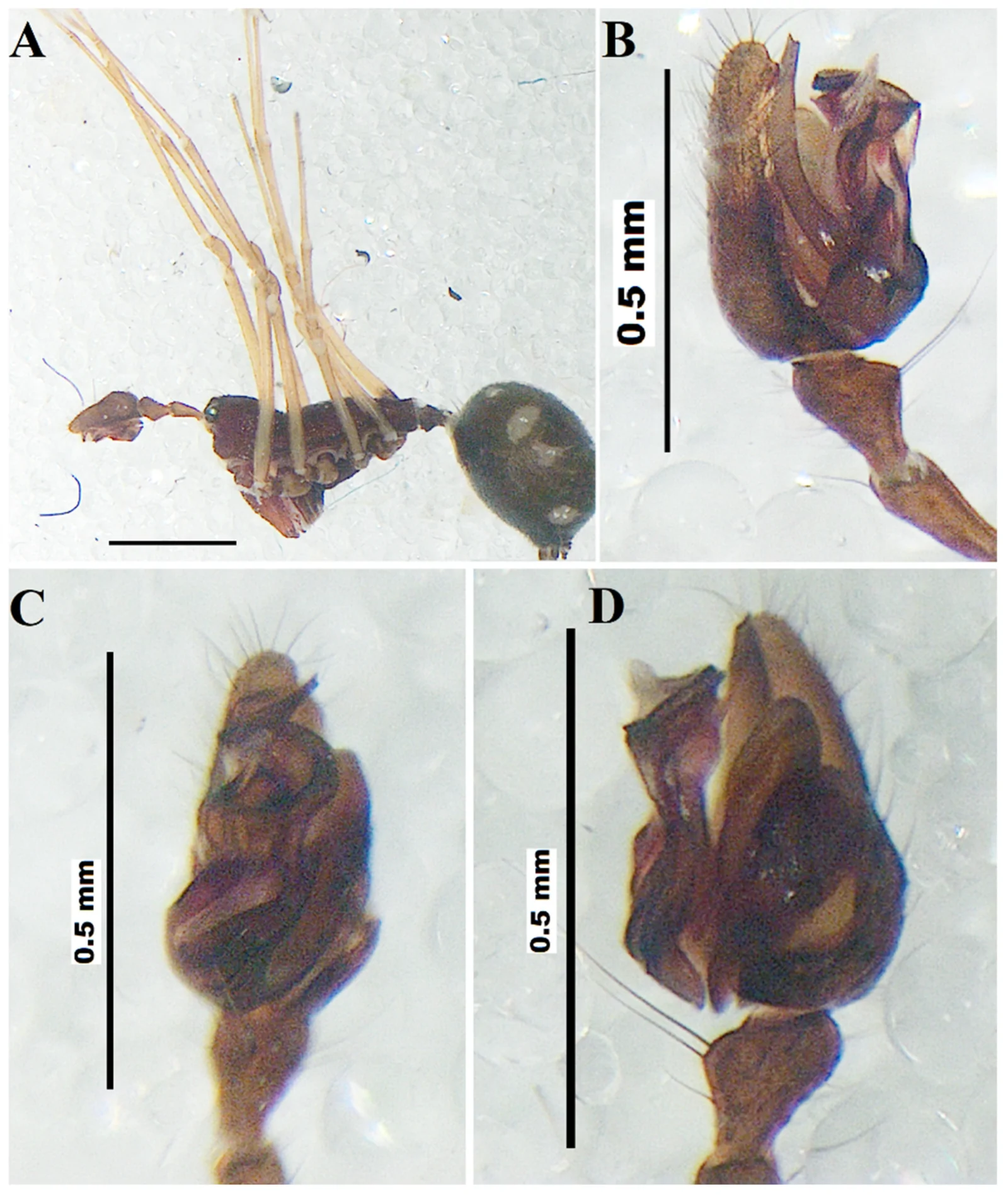
*Cresmatoneta mutinensis* (Canestrini, 1868) male from Prizren, Kosovo: (**A**) habitus, lateral view; (**B**) palp, prolateral view; (**C**) idem, ventral view; (**D**) idem, retrolateral view. Scale bars: habitus (**A**) = 1 mm; palp (**B**–**D**) = 0.5 mm.

***Cresmatoneta mutinensis* (Canestrini, 1868) (Figure 14)**

*Cresmatoneta mutinensis*: Bosselaers, 2018: 16, figs. 13, 14, 39, and 40 (mf) [22].

**Material examined.** 1 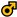 (UP Ar1030), Prizren, Prizren, 42.22216° N, 20.75338° E, 461 m, meadow, collected by hand, 30 August 2024, leg: T.H.

**Distribution.** Southern Europe, Russia (Europe), Turkey, Caucasus [1].

**Remarks.** First record of the genus for the country. The shape of the conductor and the apex of the embolus allow easy recognition.

**Figure 15:**
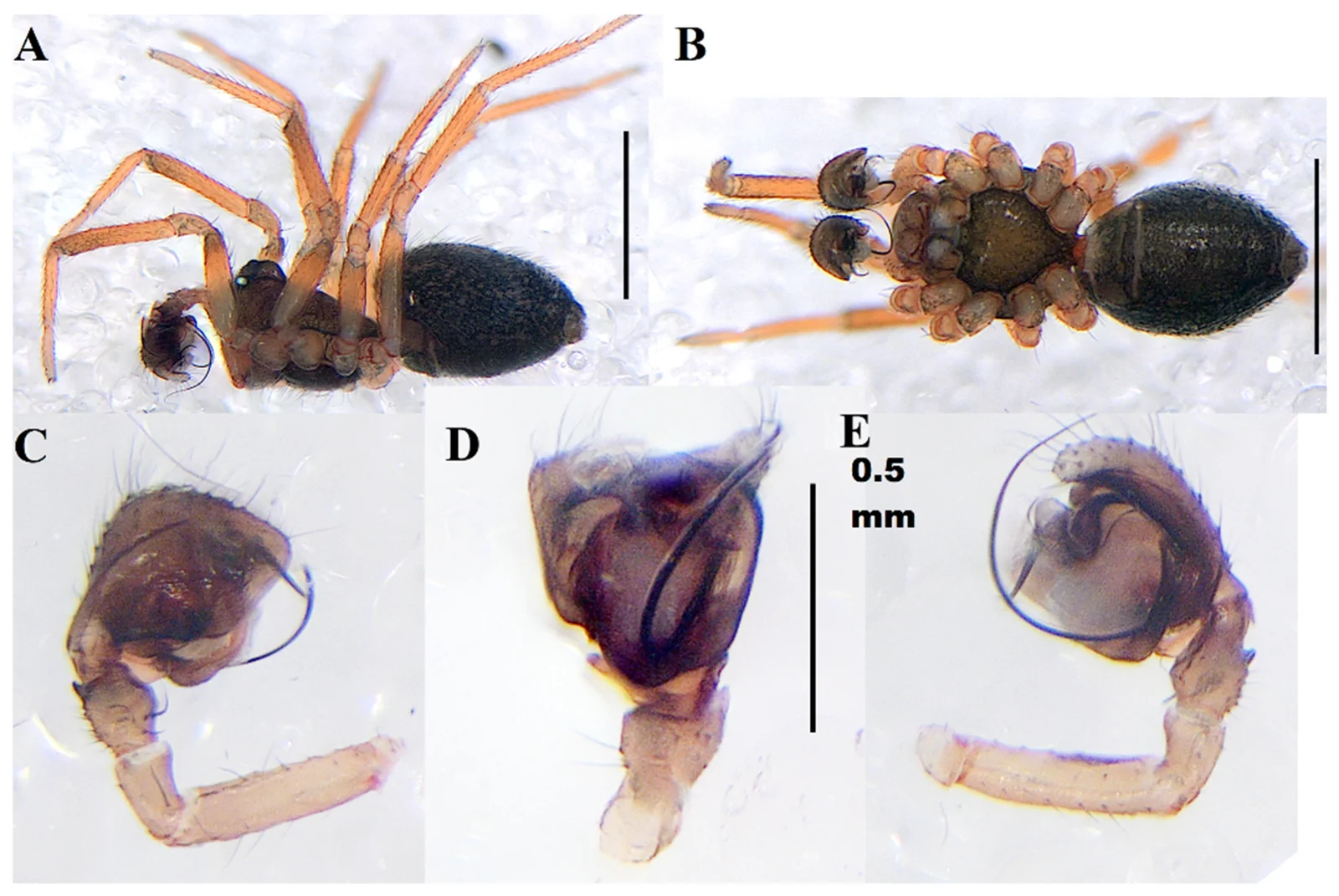
*Pocadicnemis juncea* Locket & Millidge, 1953 male from Restelicë, Kosovo: (**A**) habitus, lateral view; (**B**) idem, ventral view; (**C**) palp, retrolateral view; (**D**) idem, ventral view; (**E**) idem, prolateral view. Scale bars: habitus (**A**,**B**) = 1 mm; palp (**C**–**E**) = 0.5 mm.

***Pocadicnemis juncea* Locket & Millidge, 1953 (Figure 15)**

*Pocadicnemis juncea*: Otto & Japoshvili, 2018: 379, figs. 21 (m) [23].

**Material examined.** 1 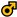 (UP Ar1031), Restelicë, Prizren, (site 2) 41.86143° N, 20.62480° E, 1660 m, valley, under rock, collected by hand, 31 May 2025, leg: A.B., D.G., D.S.

**Distribution.** Europe to Georgia [1].

**Remarks.** First record of the genus for the country. The large, sinuous embolus and wide conductor allow easy recognition of the species.

**Figure 16:**
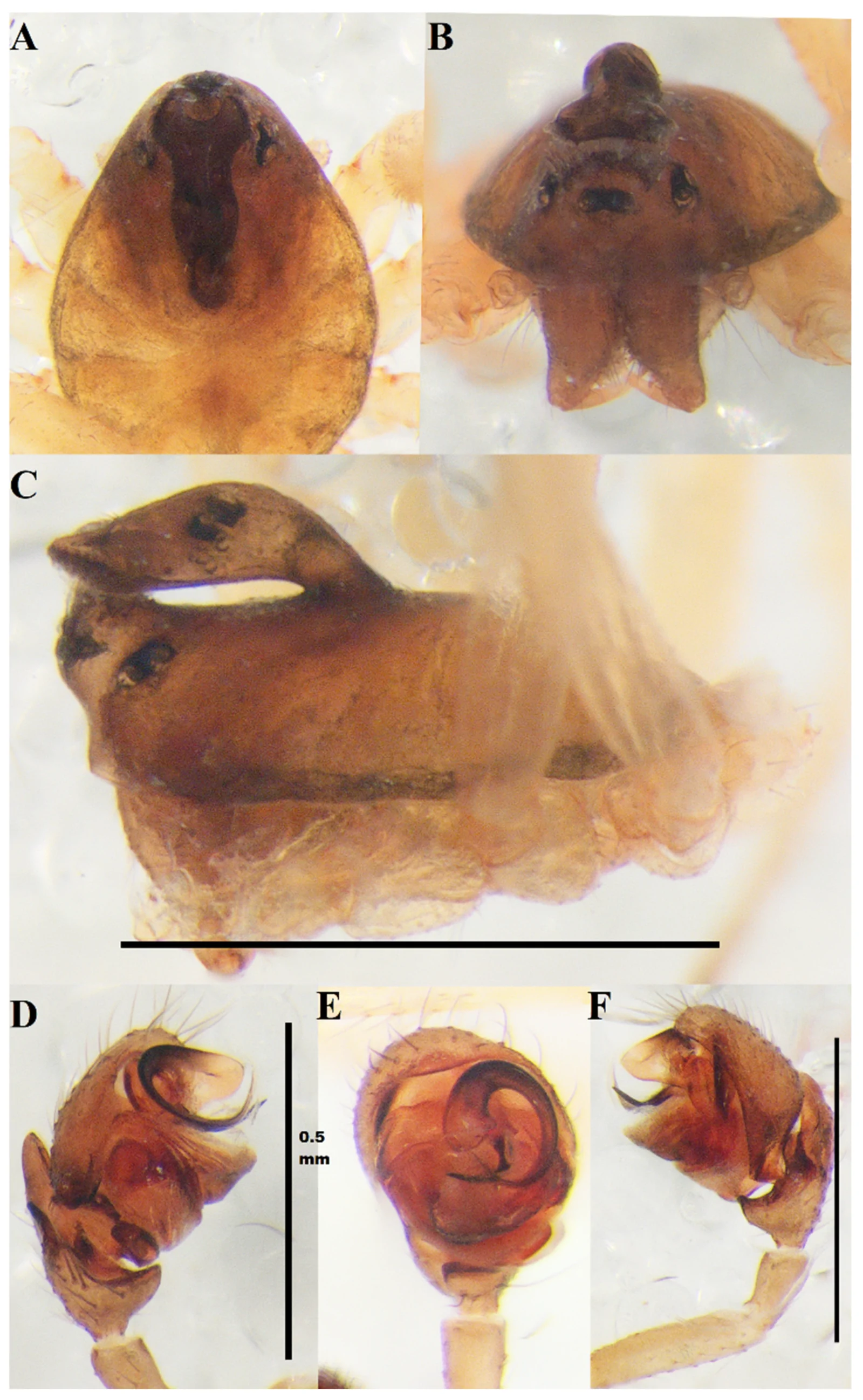
*Walckenaeria furcillata* (Menge, 1869) male from Sllatinë, Kosovo: (**A**) cephalothorax, dorsal view; (**B**) idem, frontal view; (**C**) idem, lateral view; (**D**) palp, retrolateral view; (**E**) idem, ventral view; (**F**) idem, prolateral view. Scale bars: habitus (**C**) = 1 mm; palp (**D**–**F**) = 0.5 mm.

***Walckenaeria furcillata* (Menge, 1869) (Figure 16)**

*Walckenaeria furcillata*: Danişman, 2014: 69, figs. 1A–C and 2A,B (mf) [24].

**Material examined.** 3 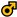

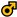
 (UP Ar1032), Sllatinë, Fushë Kosovë, 42.61349° N, 21.01908° E, 623 m, pine forest, pitfall trap, 15 October 2025, leg: D.G.

**Distribution.** Europe, Turkey, Russia (Europe to West Siberia), Iran, China, Korea, Japan [1].

**Remarks.** The cephalic modification (protuberance and invagination), distinctive position and shape of the embolus, and palpal tibial apophyses allow easy recognition of the species.

**Family Lycosidae Sundevall, 1833**

**Figure 17:**
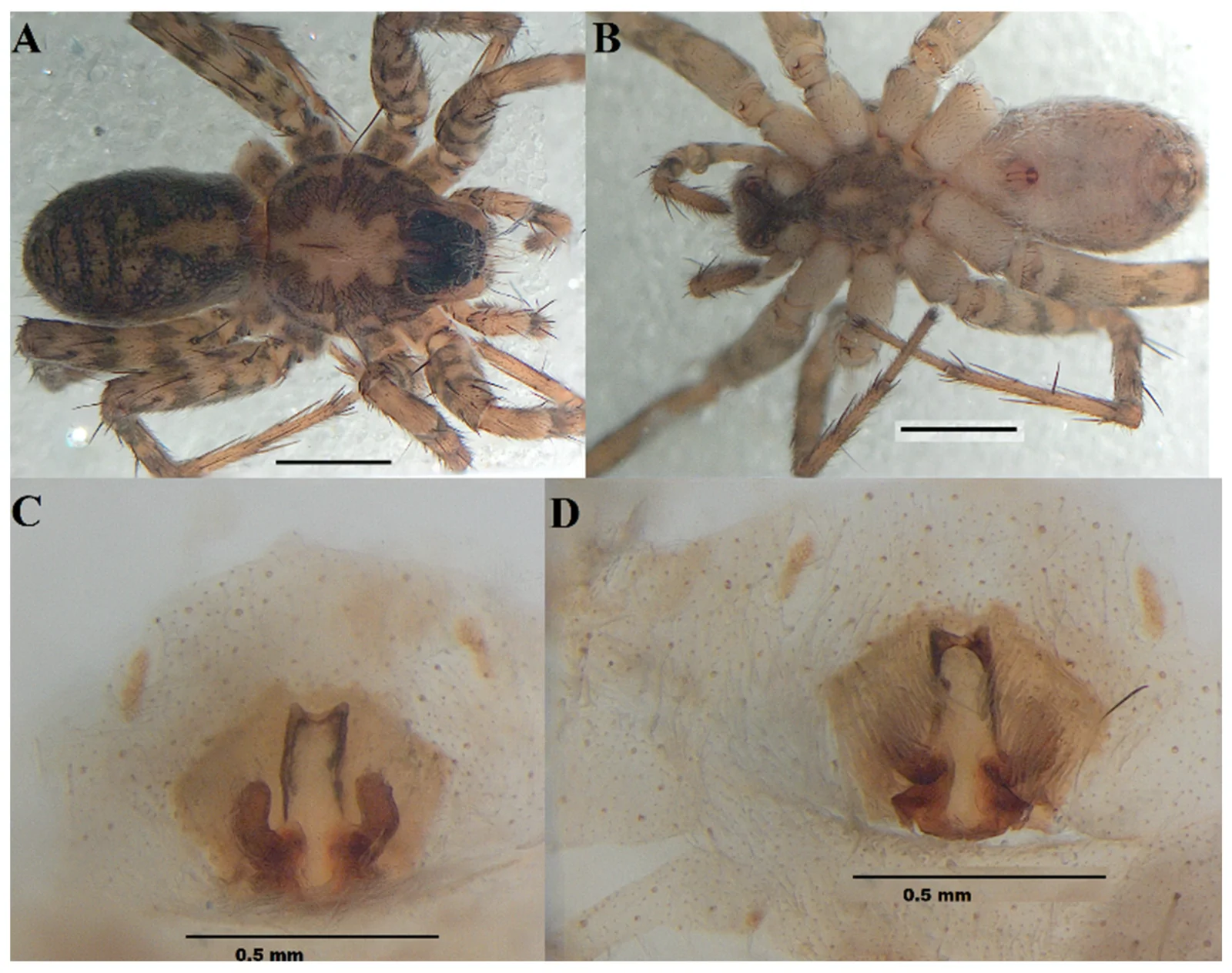
*Acantholycosa lignaria* (Clerck, 1757) female from Ksilura, Kosovo: (**A**) habitus, dorsal view; (**B**) idem, ventral view; (**C**) epigyne, dissected and cleared, ventral view; (**D**) vulva, cleared, dorsal view. Scale bars: habitus (**A**,**B**) = 1 mm; epigyne (**C**,**D**) = 0.5 mm.

***Acantholycosa lignaria* (Clerck, 1757) (Figure 17)**

*Acantholycosa lignaria*: Marusik & Omelko, 2011: 6, figs. 23, 33, 34, and 37 (mf) [25].

**Material examined.** 1 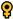 (UP Ar1033), Ksilura, Prizren, 42.19216° N, 20.78366° E, 516 m, mountain path, collected by hand, 24 August 2024, leg: T.H.

**Distribution.** Europe, Russia and China [1].

**Remarks.** First record of the genus for Kosovo. The shape of the scape, anterior hoods, and spermathecae allow easy recognition.

**Figure 18:**
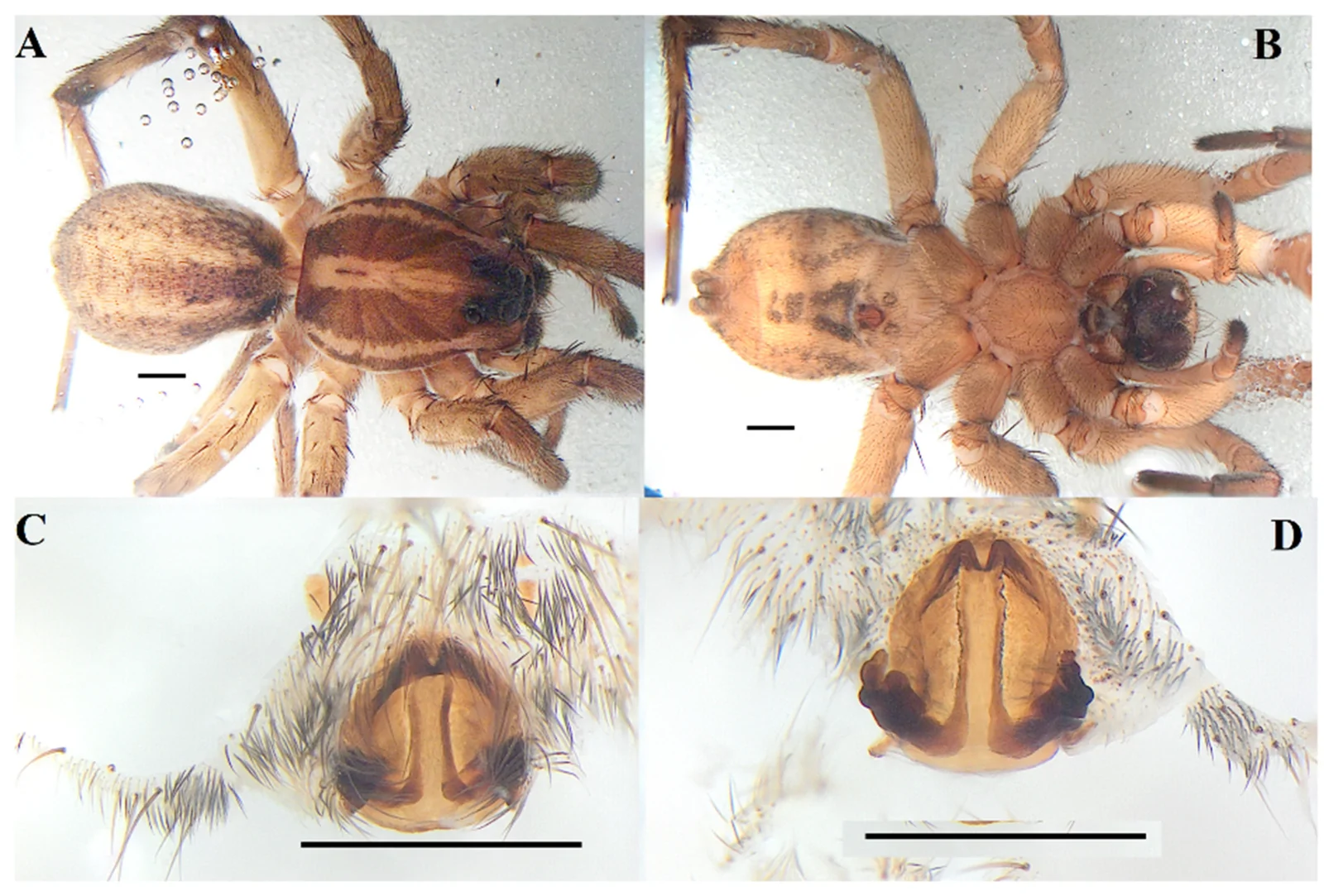
*Alopecosa fabrilis* (Clerck, 1757) female from ITP Camp, Prizren, Kosovo: (**A**) habitus, dorsal view; (**B**) idem, ventral view; (**C**) epigyne, dissected and cleared, ventral view; (**D**) vulva, cleared, dorsal view. Scale bars: 1 mm.

***Alopecosa fabrilis* (Clerck, 1757) (Figure 18)**

*Alopecosa fabrilis*: Almquist, 2006: 191, figs. 193a–g (mf) [12].

**Material examined.** 1 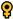 (UP Ar1034), ITP Camp, Prizren, 42.21872° N, 20.75369° E, 486 m, near buildings, collected by hand, 24 August 2024, leg: T.H.

**Distribution.** Europe, Turkey, Russia (Europe to Far East), Central Asia, China [1].

**Remarks.** The proportions of the scape and, more importantly, the shape and course of the copulatory ducts allow identification.

**Figure 19:**
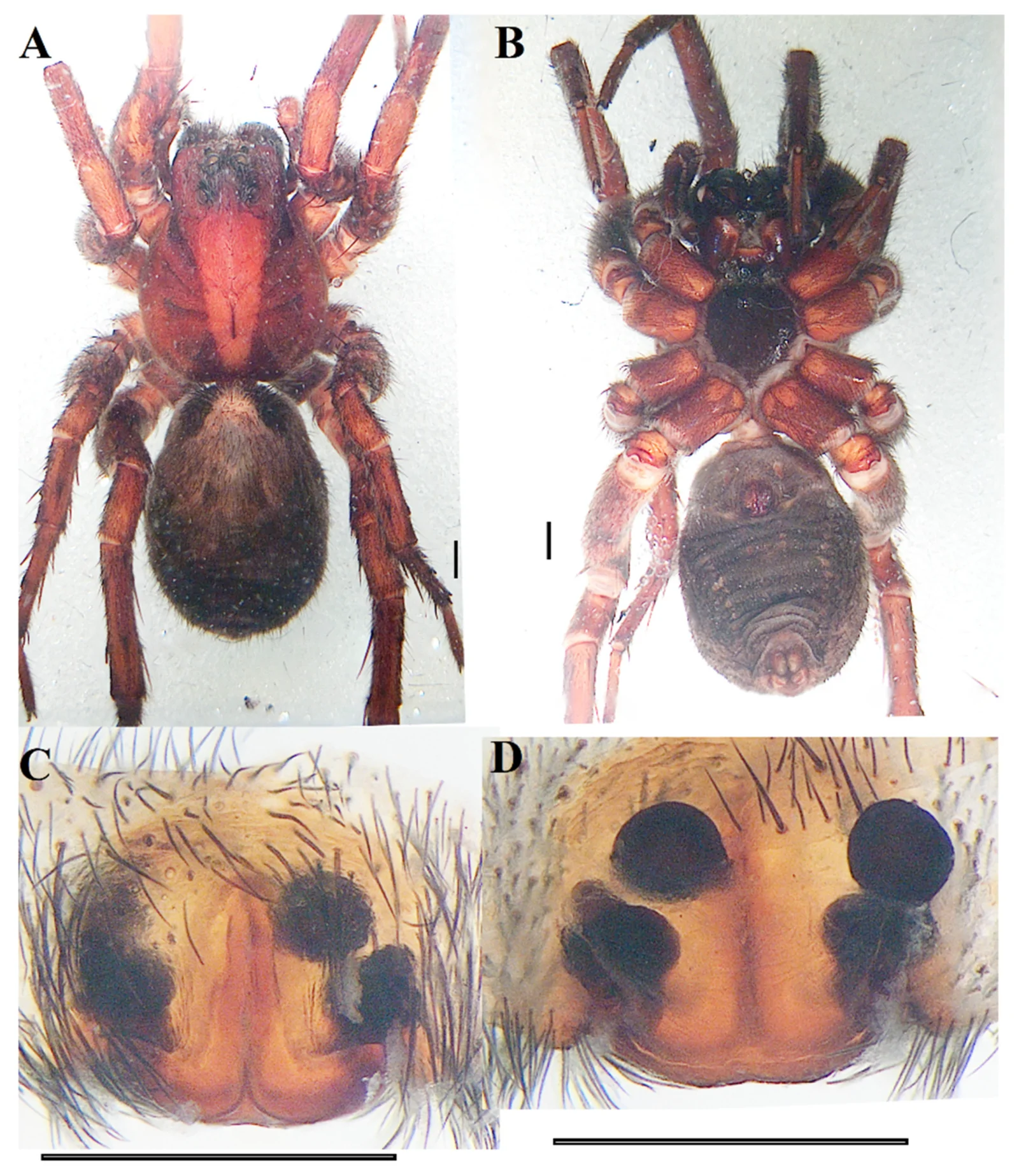
*Alopecosa inquilina* (Clerck, 1757) female from Hajle, Kosovo: (**A**) habitus, dorsal view; (**B**) idem, ventral view; (**C**) epigyne, dissected and cleared, ventral view; (**D**) vulva, cleared, dorsal view. Scale bars: 1 mm.

***Alopecosa inquilina* (Clerck, 1757) (Figure 19)**

*Alopecosa inquilina*: Almquist, 2006: 192, figs. 194a–f (mf) [12].

**Material examined.** 1 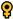 (UP Ar1035), Hajle, Pejë, 42.74680° N, 20.13838° E, 1961 m, valley, collected by hand, 25 August 2024, leg: T.H.

**Distribution.** Europe, Russia (Europe to Far East), Kazakhstan [1].

**Remarks.** The wide scape with inverse Y-shaped indentation in the posterior quarter allows recognition of this species.

**Figure 20:**
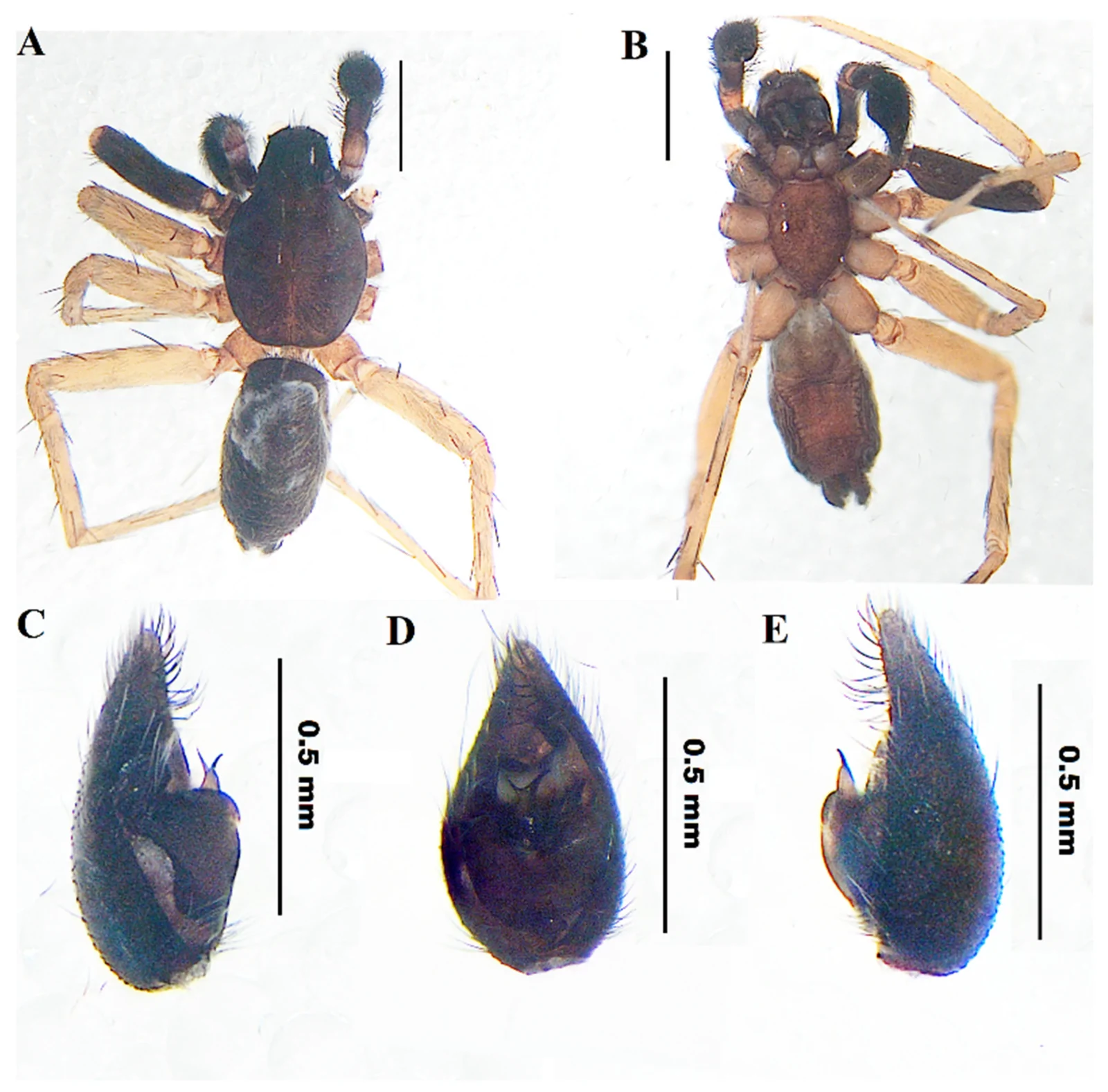
*Aulonia albimana* (Walckenaer, 1805) male from Gorancë, Kosovo: (**A**) habitus, dorsal view; (**B**) idem, ventral view; (**C**) cymbium (dissected), prolateral view; (**D**) idem, ventral view; (**E**) idem, retrolateral. Scale bars: habitus (**A**,**B**) = 1 mm; palp (**C**–**E**) = 0.5 mm.

***Aulonia albimana* (Walckenaer, 1805) (Figure 20)**

*Aulonia albimana*: Almquist, 2006: 206, figs. 207a–f (mf) [12].

**Material examined.** 1 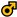 (UP Ar1036), Gorancë, Hani i Elezit, 42.12828° N, 21.24977° E, 699 m, rocky escarpment, collected by hand, 30 May 2025, leg: D.G., D.S.; 1 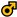 (UP Ar1037), Debëlldëh, Viti, (site 2) 42.25023° N, 21.40215° E, 999 m, forest, collected with aspirator, 20.06.2020, leg: A.B., D.G., M.M.

**Distribution.** Europe, Turkey, and the Caucasus [1].

**Remarks.** First record of the genus for the country. The asymmetrical Y-shaped bifurcate embolus allows identification.

**Figure 21:**
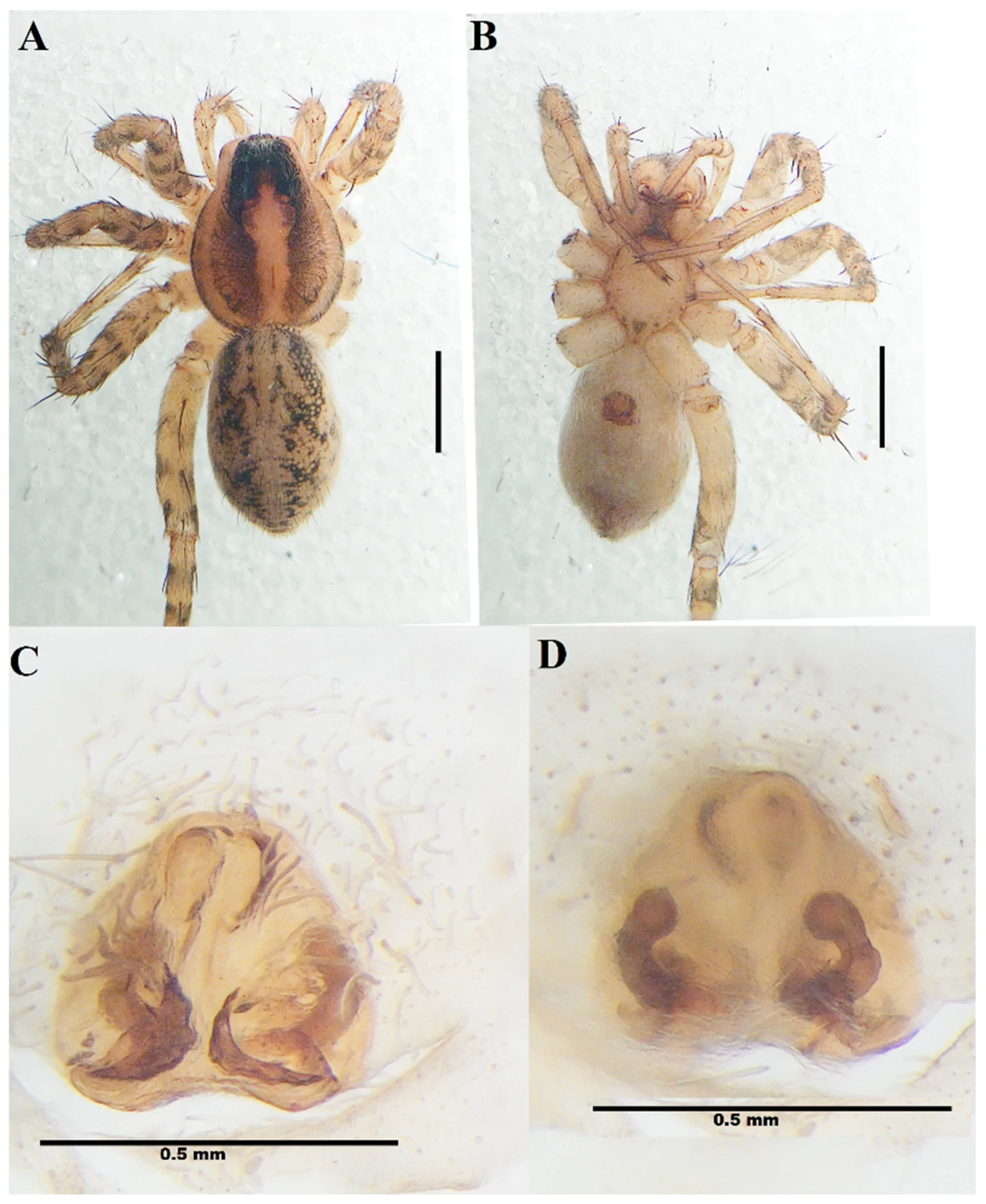
*Pardosa roscai* (Roewer, 1951) female from Landovicë, Kosovo: (**A**) habitus, dorsal view; (**B**) idem, ventral view; (**C**) epigyne, *dissected* and cleared, ventral view; (**D**) vulva, cleared, dorsal view. Scale bars: habitus (**A**,**B**) = 1 mm; epigyne (**C**,**D**) = 0.5 mm.

***Pardosa roscai* (Roewer, 1951) (Figure 21)**

*Pardosa roscai*: Bayram, Efil & Deltshev, 2009: 465, figs. 1–5 (mf) [26].

**Material examined.** 1 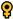 (UP Ar1038), Landovicë, Prizren, 42.27816° N, 20.66583° E, 357 m, meadow, collected by hand, 25 August 2024, leg: T.H.

**Distribution.** Bulgaria, Romania, Cyprus, Turkey, Iraq, Iran [1].

**Remarks.** We initially considered this material to be *Pardosa cribrata* Simon, 1876, but comparison against [26] showed that the proportions of the spermathecae and its biogeography are more congruent with *P. roscai*. We note these species are morphologically close in the female and that only one suboptimal illustration of the *P. cribrata* female vulva currently exists.

**Figure 22:**
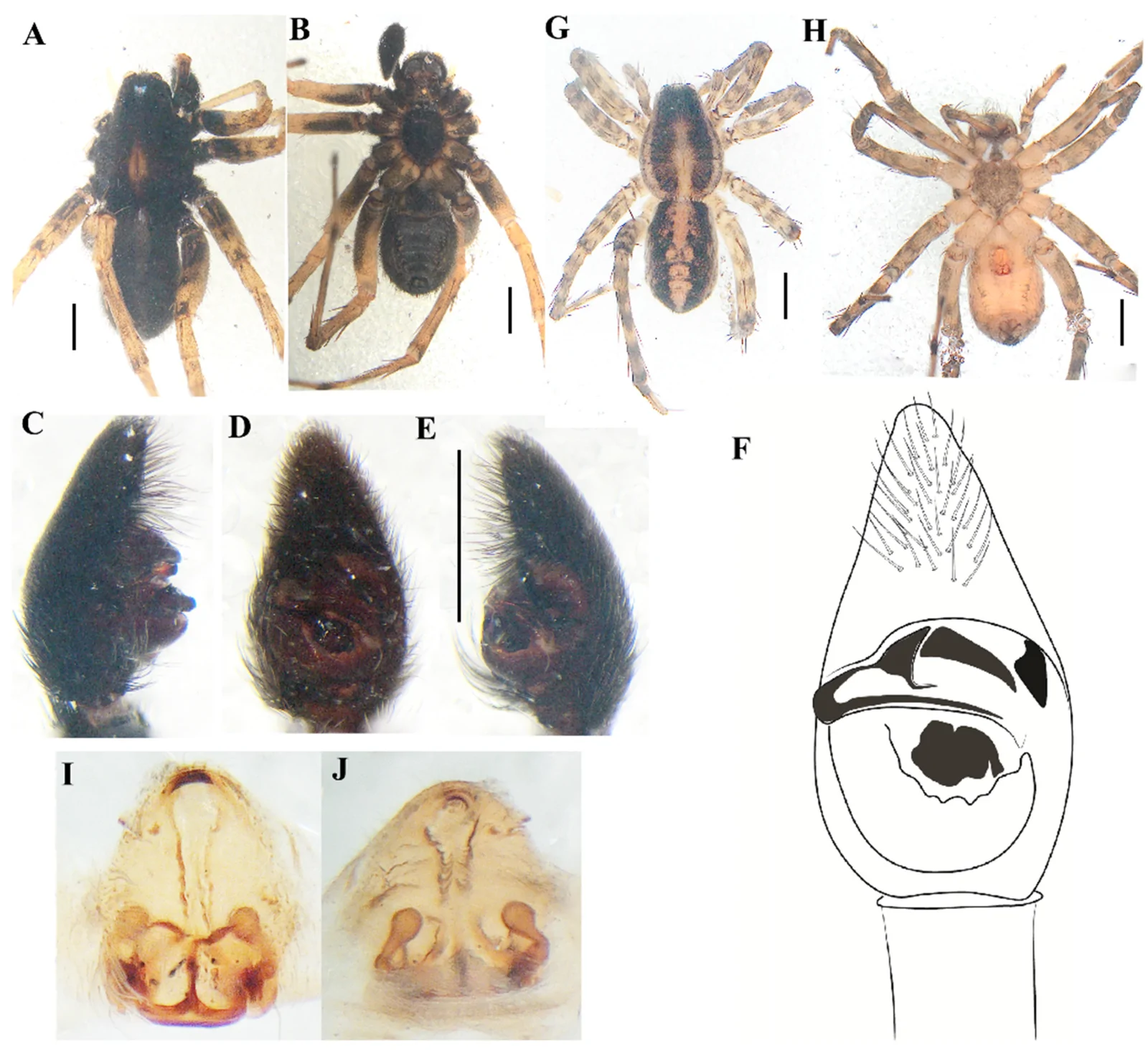
*Pardosa vittata* (Keyserling, 1863) male and female from Henc, Kosovo: (**A**) male habitus, dorsal view; (**B**) idem, ventral view; (**C**) male palp, prolateral view; (**D**) idem, ventral view; (**E**) idem, retrolateral view; (**F**) idem, drawing of ventral view; (**G**) female, habitus, dorsal view; (**H**) idem, ventral view; (**I**) epigyne, dissected and cleared, ventral view; (**J**) vulva, cleared, dorsal view. Scale bars: 1 mm.

***Pardosa vittata* (Keyserling, 1863) (Figure 22)**

*Pardosa vittata* Thaler, 1987: 194, figs. 1–5 (mf) [27].

**Material examined.** 1 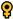 (UP Ar1039), Henc, Fushë Kosovë, 42.58227° N, 21.04886° E, 536 m, wetland, collected by hand, 26 August 2024, leg: T.H.; 3 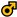 (UP Ar1040), Vërmicë, Prizren, 42.15418° N, 20.53370° E, 282 m, wetland, collected by hand, 1 June 2025, leg: A.B., D.G., D.S.

**Distribution.** Europe to Georgia [1].

**Remarks.** The morphology of the embolus, median apophysis, scape and spermathecae are all congruent for this species.

**Figure 23:**
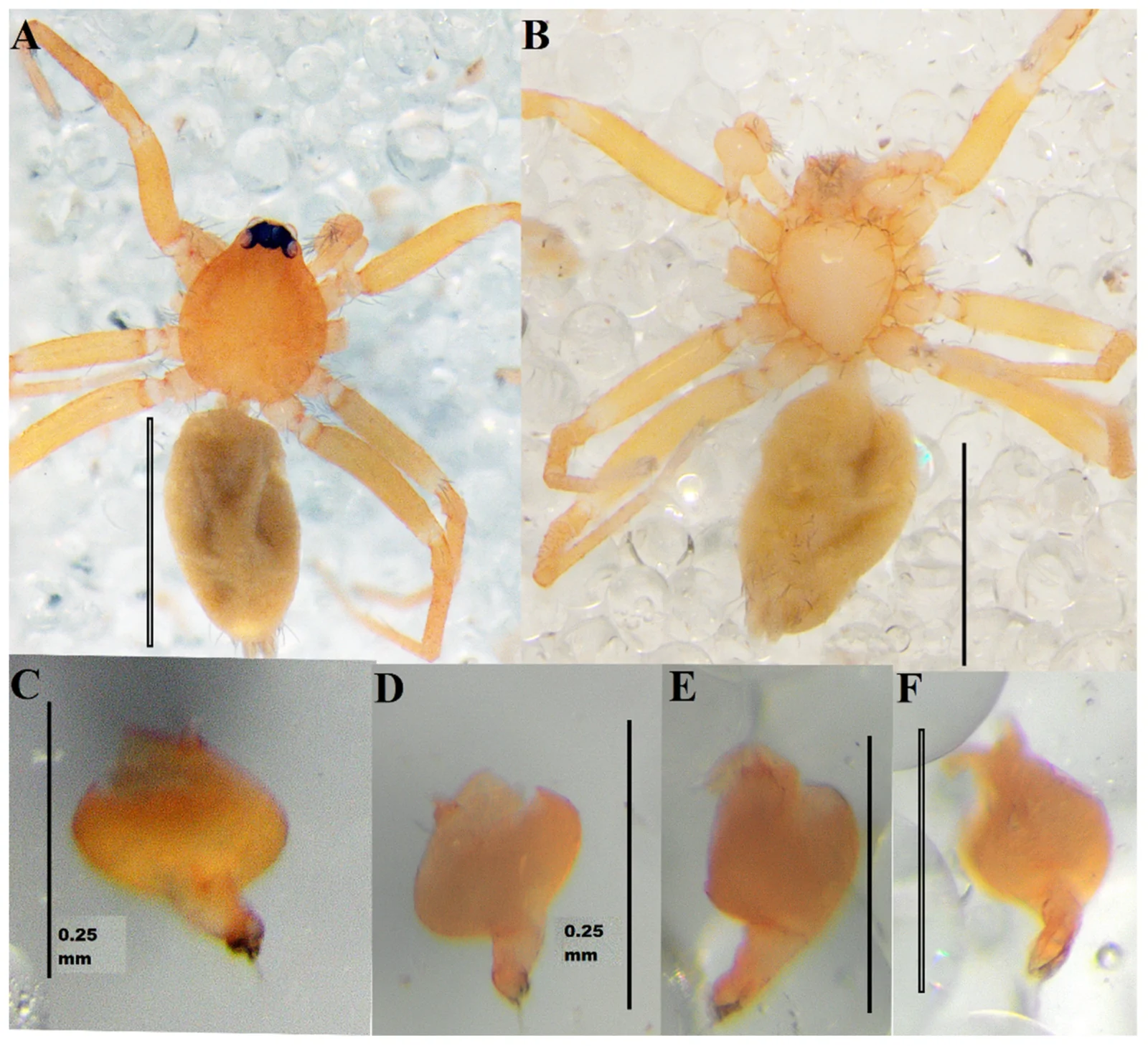
*Orchestina pavesii* (Simon, 1873) male from Bajgorë, Kosovo: (**A**) habitus, dorsal view; (**B**) idem, ventral view; (**C**) palpal bulb (dissected), dorsal view; (**D**) idem, ventral view; (**E**) idem, retrolateral view; (**F**) idem, apical–prolateral view. Scale bars: habitus (**A**,**B**) = 1 mm; palp (**C**–**F**) = 0.25 mm.

**Family Oonopidae Simon, 1890 *Orchestina pavesii* (Simon, 1873) (Figure 23)**

*Orchestina pavesii*: Le Peru, 2011: 306, fig. 532 (mf) [28].

**Material examined.** 1 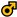 (UP Ar1041), Bajgorë, Mitrovicë, 42.96002° N, 21.01451° E, 1252 m, beech forest, collected with leaflitter sieve, 10 May 2026, leg: D.G.

**Distribution.** Canary Islands, Southwest Europe to Greece, Bulgaria, Algeria, Egypt, Yemen. Introduced to Japan [1].

**Remarks.** First record of the family and genus for the country. The morphology of the embolus, including its shape and keelation, allows recognition of the species.

**Family Oxyopidae Thorell, 1869**

**Figure 24:**
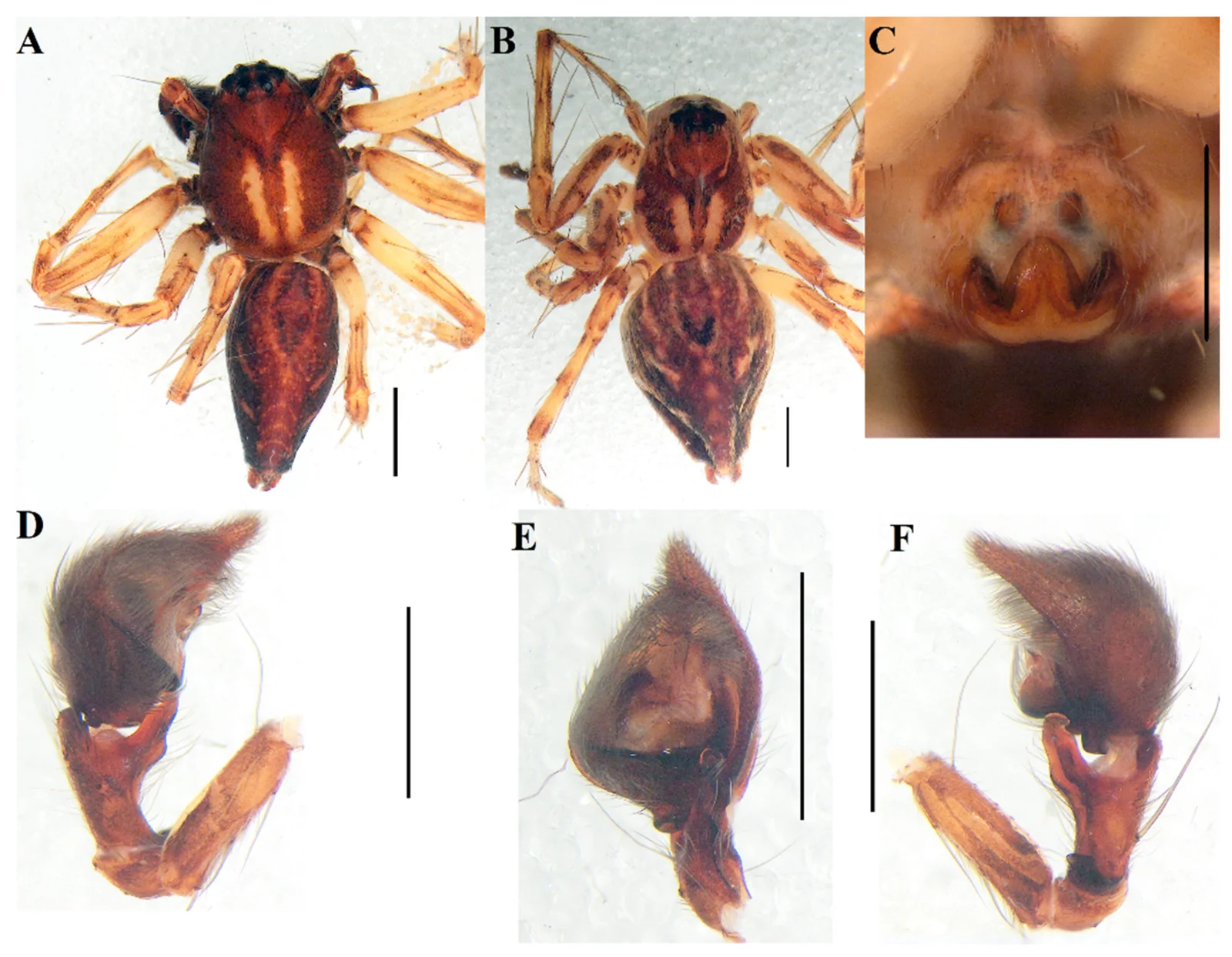
*Oxyopes heterophthalmus* (Latreille, 1804) male and female from Grabanicë, Kosovo: (**A**) male, habitus, dorsal view; (**B**) female, habitus, dorsal view; (**C**) female epigyne, undissected, ventral view; (**D**) male palp, prolateral view; (**E**) idem, ventral view; (**F**) idem, retrolateral view. Scale bars: 1 mm.

***Oxyopes heterophthalmus* (Latreille, 1804) (Figure 24)**

*Oxyopes heterophthalmus* Esyunin & Tuneva, 2009: 165, figs. 1.1–2, 2.2, 5, 3.1, 4.3–4 (mf) [29].

**Material examined.** 2 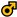

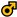
, 3 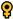

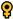
 (UP Ar1042), Grabanicë, Klinë, 42.58309° N, 20.55325° E, 370 m, agricultural land, collected with butterfly net, 15 August 2025, leg: M.M.

**Distribution.** Europe, North Africa to Middle East, Turkey, Caucasus, Kazakhstan, China [1].

**Remarks.** Male left palp has a broken tibial apophysis, a phenomenon mostly recently discussed by Tchilinguirian & Sherwood [30]. The morphology of the male palpal sclerites and female scape, combined with the habitus, allows easy recognition of this common species.

**Figure 25:**
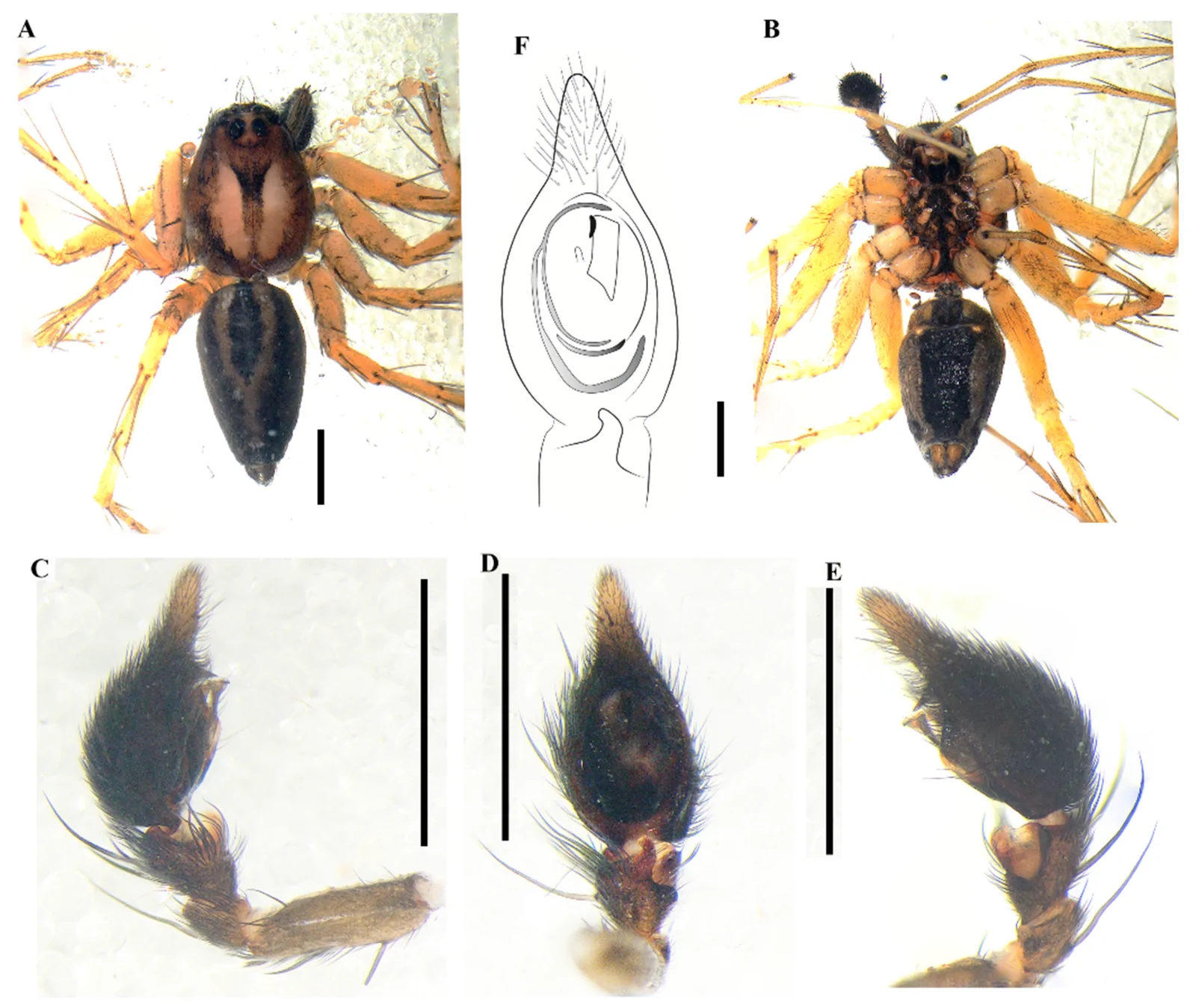
*Oxyopes nigripalpis* Kulczyński, 1891 male from Vërmicë, Kosovo: (**A**) male habitus, dorsal view; (**B**) idem, ventral view; (**C**) male palp, prolateral view; (**D**) idem, ventral view; (**E**) idem, retrolateral view; (**F**) idem, drawing of ventral view. Scale bars: 1 mm.

***Oxyopes nigripalpis* Kulczyński, 1891 (Figure 25)**

*Oxyopes nigripalpis*: Naumova, Blagoev & Deltshev, 2021: 242, figs. 14A–D (m) [31].

**Material examined.** 2 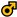

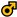
 (UP Ar1043), Vërmicë, Prizren, 42.15418° N, 20.53370° E, 282 m, wetland, collected by hand, 2 June 2025, leg: A.B., D.G., D.S.

**Distribution.** Mediterranean [1].

**Remarks.** The shape of the retrolateral tibial apophysis in conjunction with the embolus and conductor allows easy recognition.

**Family Philodromidae Thorell, 1869**

**Figure 26:**
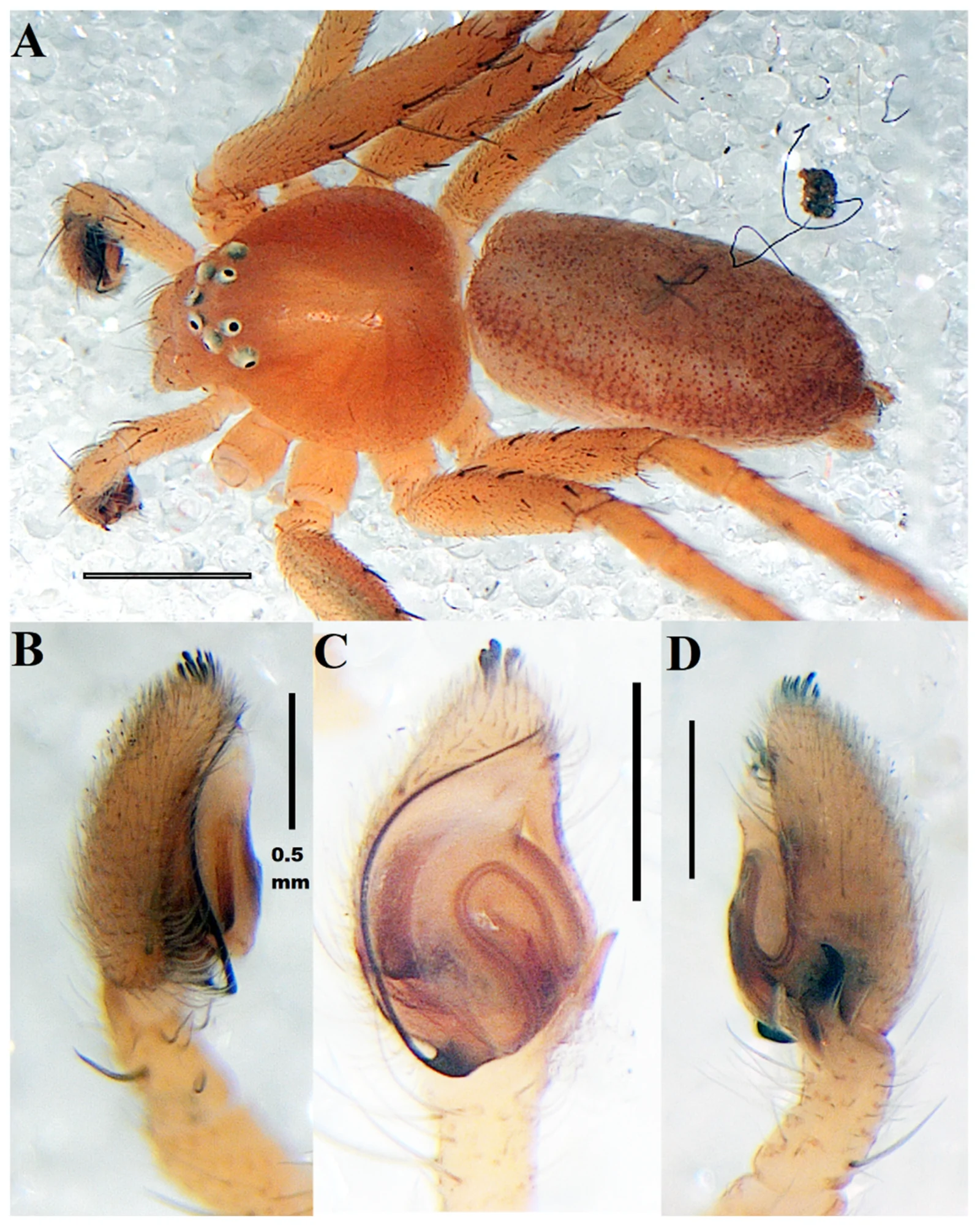
*Philodromus albidus* Kulczyński, 1911 male from Shkugëz, Kosovo: (**A**) habitus, dorsal view; (**B**) palp, prolateral view; (**C**) idem, ventral view; (**D**) idem, retrolateral view. Scale bars: habitus (**A**) = 1 mm; palp (**B**–**D**) = 0.5 mm.

***Philodromus albidus* Kulczyński, 1911 (Figure 26)**

*Philodromus albidus*: Kastrygina & Kovblyuk, 2016: 44, figs. 1–3, 7, 8, 11, and 12 (mf) [32].

**Material examined.** 1 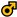 (UP Ar1044), Shkugëz, Gjakovë, 42.35989° N, 20.42165° E, 375 m, forest, collected by vegetation beating, 12 May 2025, leg: D.G.

**Distribution.** Europe, Turkey, and the Caucasus [1].

**Remarks.** The shape of the tegulum (with its posterior half, before the sperm duct, accounting for more than half of the tegulum’s width), in conjunction with the distally thicker retrolateral apophysis, separates this species from the closely related *P. rufus* Walckenaer, 1826.

**Figure 27:**
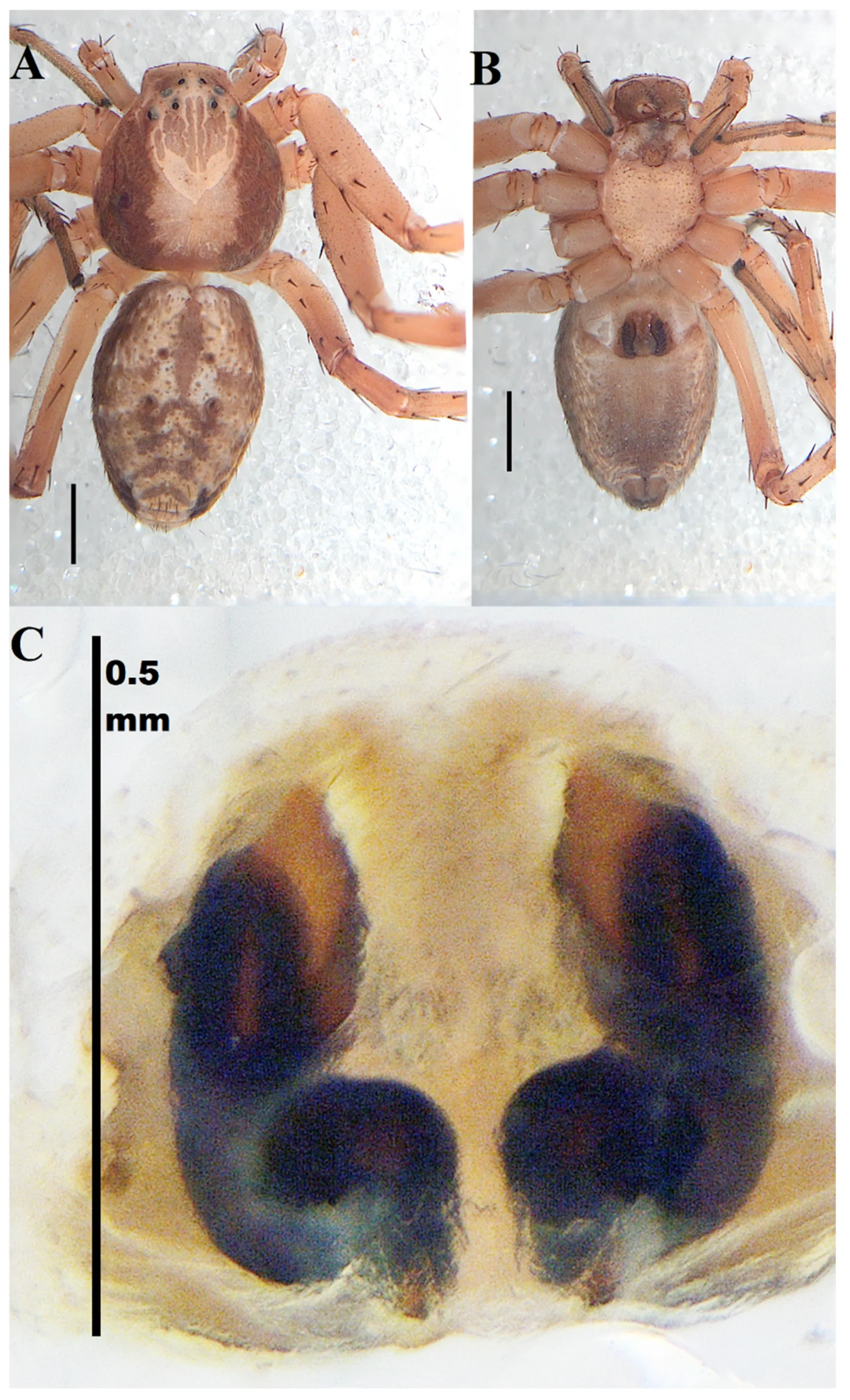
*Philodromus praedatus* O. Pickard-Cambridge, 1871 female from Prevall, Kosovo: (**A**) habitus, dorsal view; (**B**) idem, ventral view; (**C**) vulva, cleared, dorsal view. Scale bars: habitus (**A**,**B**) = 1 mm; epigyne (**C**) = 0.5 mm.

***Philodromus praedatus* O. Pickard-Cambridge, 1871 (Figure 27)**

*Philodromus praedatus*: Roberts, 1993: 8, figs. 3b and 4b (f) [33].

**Material examined.** 1 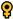 (UP Ar1045), Prevall, Prizren, 42.17529° N, 20.97426° E, 1373 m, meadow, collected by vegetation beating, 30 June 2025, leg: D.G., H.I.

**Distribution.** Europe, Russia (Europe to South Siberia), Georgia, Azerbaijan, Iran [1].

**Remarks.** The basally sinuous spermathecae allow recognition of this species.

**Figure 28:**
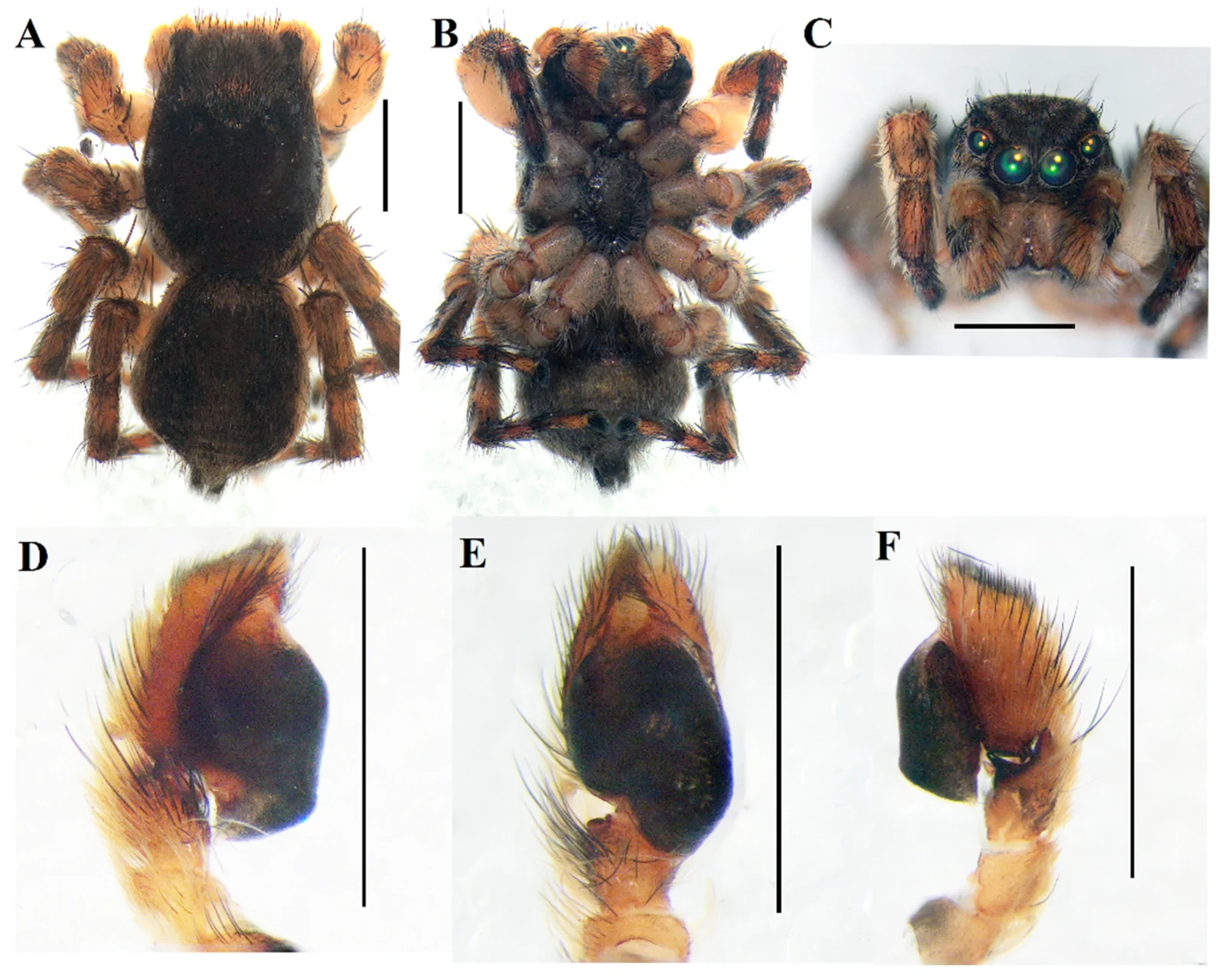
*Aelurillus v-insignitus* (Clerck, 1757) male from Debëlldëh, Kosovo: (**A**) habitus, dorsal view; (**B**) idem, ventral view; (**C**) idem, frontal view; (**D**) palp, prolateral view; (**E**) idem, ventral view; (**F**) idem, retrolateral view. Scale bars: habitus (**A**–**C**) = 1 mm; palp (**D**–**F**) = 0.5 mm.

**Family Salticidae Blackwall, 1841 *Aelurillus v-insignitus* (Clerck, 1757) (Figure 28)**

*Aelurillus v-insignitus*: Marta et al., 2024: 8, Figures 2A and 3A (mf) [34].

**Material examined.** 1 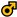 (UP Ar1046), Debëlldëh, Viti, (site 3) 42.25071° N, 21.40313° E, 1032 m, meadow, collected with aspirator, 25 April 2026, leg: A.B., D.G., M.M.

**Distribution.** Europe, Turkey, Caucasus, Russia (Europe to Far East), Kazakhstan, Central Asia, China [1].

**Remarks.** First record of the genus for the country. The shape of the retrolateral tibial apophysis and embolus allows recognition of the species.

**Figure 29:**
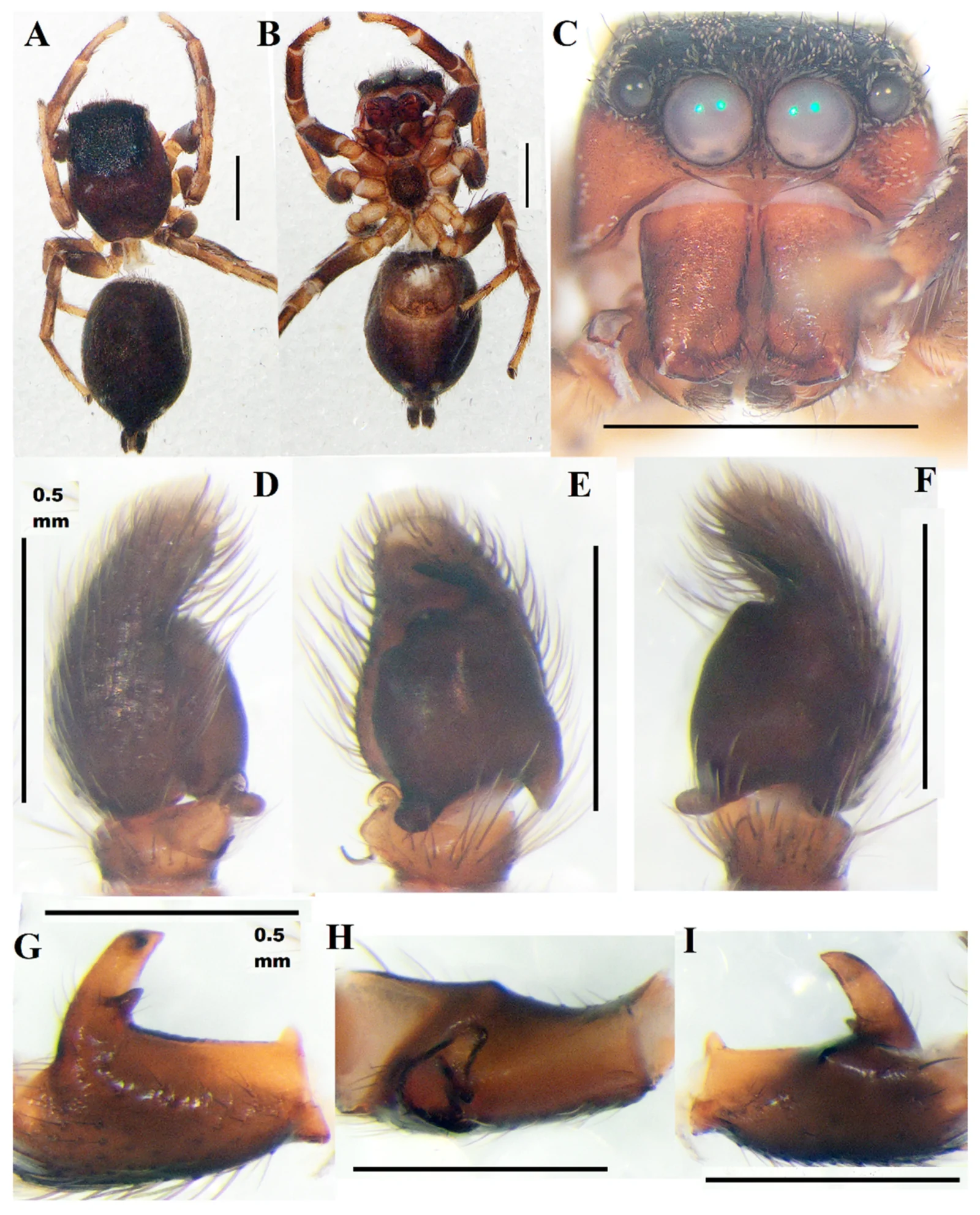
*Heliophanus tribulosus* Simon, 1868 male from Lumbardhi i Prizrenit, Kosovo: (**A**) habitus, dorsal view; (**B**) idem, ventral view; (**C**) cephalothorax, frontal view, close-up; (**D**) palp, prolateral view; (**E**) idem, ventral view; (**F**) idem, retrolateral view; (**G**) palpal femora, showing apophysis, prolateral view; (**H**) idem, ventral view; (**I**) idem, retrolateral view. Scale bars: habitus (**A**–**C**) 1 mm; palp (**D**–**F**) = 0.5 mm; palpal femora (**G**–**I**) = 0.5 mm.

***Heliophanus tribulosus* Simon, 1868 (Figure 29)**

*Heliophanus tribulosus*: Metzner, 1999: 100, figs. 65a–j [35].

**Material examined.** 1 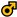 (UP Ar1047), Lumbardhi i Prizrenit, 42.20652° N, 20.75558° E, 441 m, valley, collected with aspirator, 24 August 2024, leg: T.H.

**Distribution.** Europe to Kazakhstan [1].

**Remarks.** The morphology of the femoral apophysis, tegular apophyses, and embolus is congruent for this taxon.

**Figure 30:**
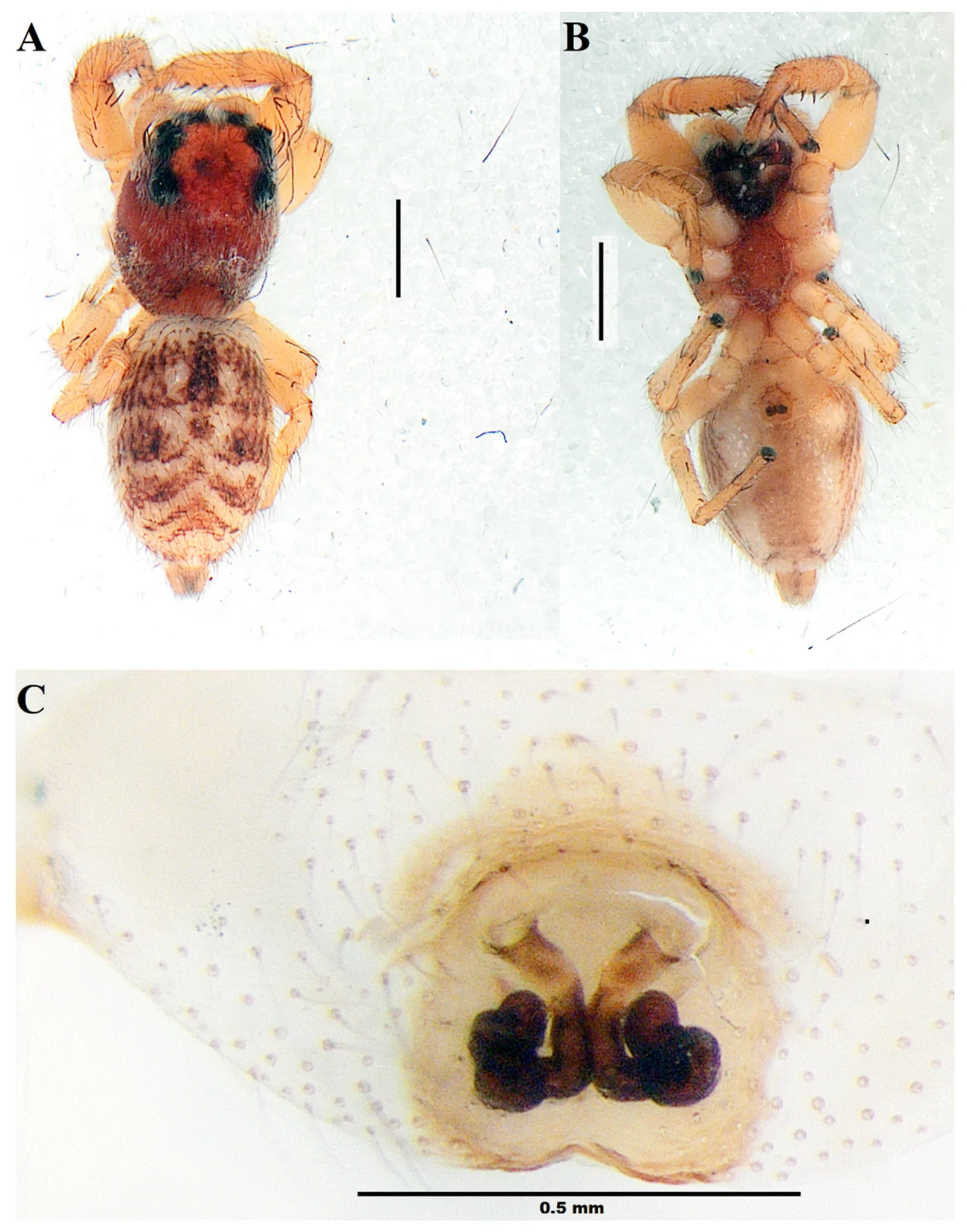
*Macaroeris nidicolens* (Walckenaer, 1802) female from ITP Camp, Prizren, Kosovo: (**A**) habitus, dorsal view; (**B**) idem, ventral view; (**C**) vulva, cleared, dorsal view. Scale bars: habitus (**A**,**B**) = 1 mm; epigyne (**C**) = 0.5 mm.

***Macaroeris nidicolens* (Walckenaer, 1802) (Figure 30)**

*Macaroeris nidicolens*: Logunov & Schäfer, 2025: 404, figs. 1–17, 146–149, 153–159, 164, 165, 167–169, and 171–190 (mf) [36].

**Material examined.** 1 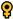 (UP Ar1048), ITP Camp, Prizren, 42.21872° N, 20.75369° E, 486 m, near building, collected by hand, 24 August 2024, leg: T.H.

**Distribution.** Europe, North Africa to Turkey, Caucasus, Turkmenistan, Iran. Introduced to Sri Lanka [1].

**Remarks.** First record of the genus for the country. The tube-like copulatory openings and ducts allow easy recognition of this species.

**Figure 31:**
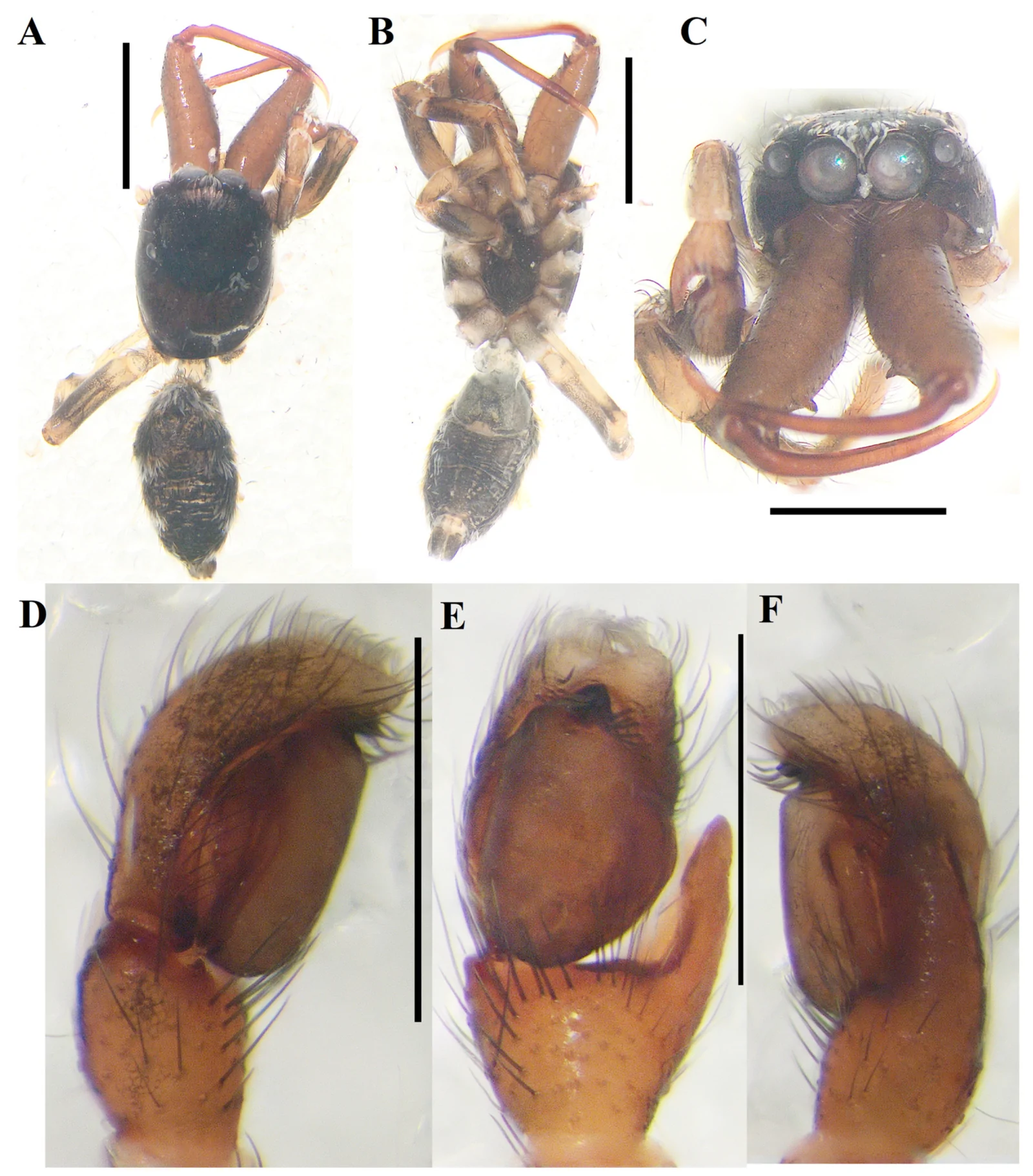
*Salticus unicolor* (Simon, 1868) male from Obiliq, Kosovo: (**A**) habitus, dorsal view; (**B**) idem, ventral view; (**C**) cephalothorax, frontal view, close-up; (**D**) palp, prolateral view; (**E**) idem, ventral view; (**F**) idem, retrolateral view. Scale bars: habitus (**A**–**C**) = 1 mm; palp (**D**–**F**) = 0.5 mm.

***Salticus unicolor* (Simon, 1868) (Figure 31)**

*Salticus unicolor*: Schäfer, 2021: 90, figs. 12a–b, 13a–b, 16a–b, 17b, 18a–b, 19a–c (mf) [37].

**Material examined.** 1 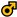 (UP Ar1049), Obiliq, 42.66431° N, 21.10967° E, 557 m, agricultural land, collected by hand, 25 August 2025, leg: D.G., H.I.

**Distribution.** Bulgaria, Greece, Hungary, and Slovakia [1].

**Remarks.** The short embolus, wide and blunted retrolateral tibial apophysis, and cheliceral morphology, allow assignment of this species to *S. unicolor* and differentiate it from the closely related *S. zebraneus* (C. L. Koch, 1837).

**Figure 32:**
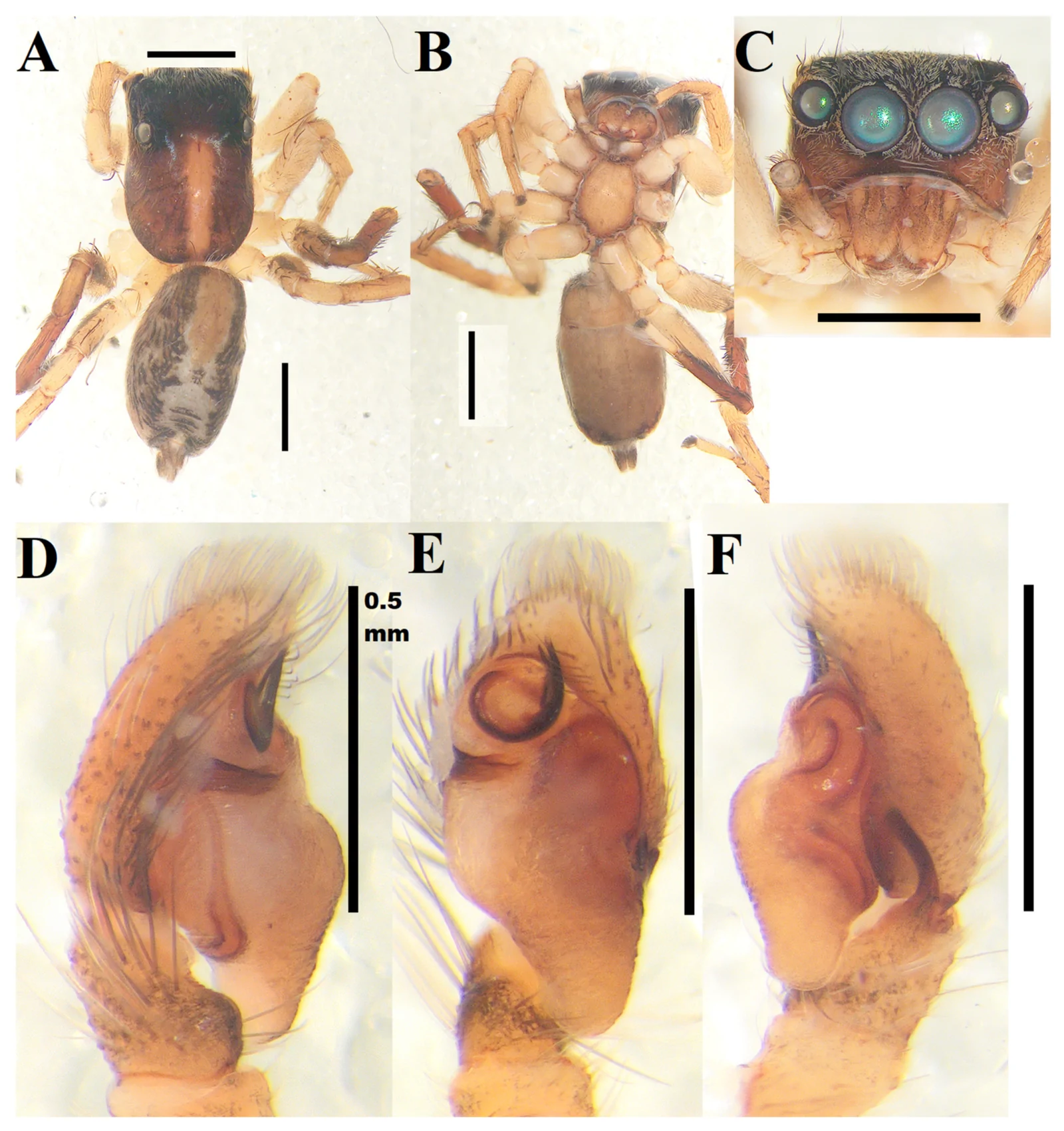
*Saitis tauricus* Kulczyński, 1905 male from Taukbashqe, Kosovo: (**A**) habitus, dorsal view; (**B**) idem, ventral view; (**C**) cephalothorax, frontal view, close-up; (**D**) palp, prolateral view; (**E**) idem, ventral view; (**F**) idem, retrolateral view. Scale bars: habitus (**A**–**C**) = 1 mm; palp (**D**–**F**) = 0.5 mm.

***Saitis tauricus* Kulczyński, 1905 (Figure 32)**

*Saitis tauricus*: Seropian et al., 2023: 278, figs. 111 and 112 (m) [38].

**Material examined.** 1 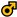 (UP Ar1050), Taukbashqe, Prishtinë, 42.66419° N, 21.17338° E, 614 m, urban park, collected with aspirator, 11 April 2026, leg: D.G.

**Distribution.** Italy, Hungary, Romania, Ukraine, North Macedonia, Bulgaria, Greece, Turkey, Georgia [1].

**Remarks.** First record of the genus for the country. The spiral, bifurcate embolus and elongate digitiform retrolateral apophsis allow easy recognition of the species.

**Family Tetragnathidae Menge, 1866**

**Figure 33:**
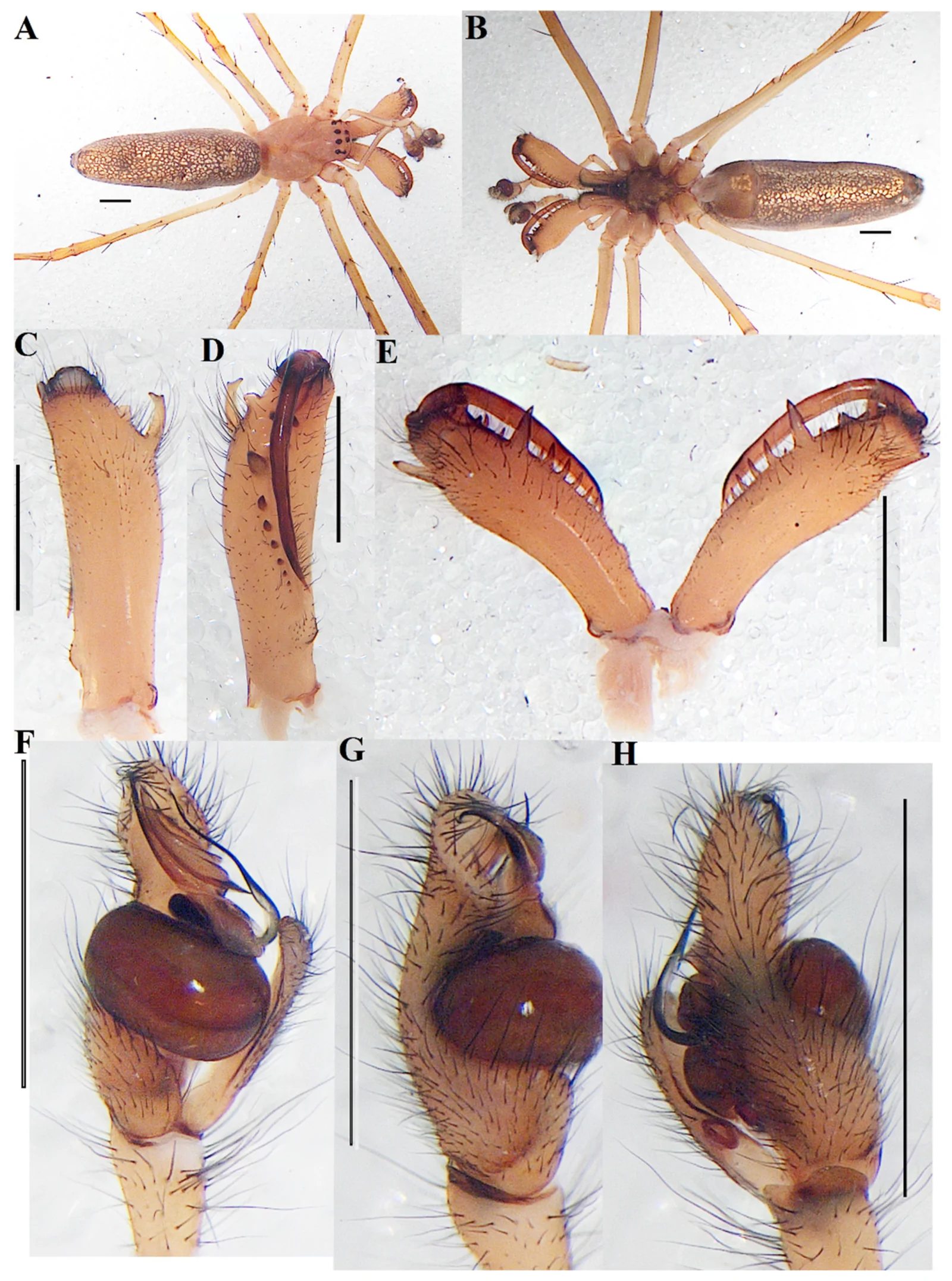
*Tetragnatha montana* Simon, 1874 male from Dush, Kosovo: (**A**) habitus, dorsal view; (**B**) idem, ventral view; (**C**) right chelicera, dorsal view; (**D**) idem, ventral view; (**E**) both chelicerae, dorsal view; (**F**) palp, ventral view; (**G**) idem, prolateral view; (**H**) idem, dorsal view. Scale bars: 1 mm.

***Tetragnatha montana* Simon, 1874 (Figure 33)**

*Tetragnatha montana*: Morano, 2020: 112, figs. 75–80 (mf) [39].

**Material examined.** 1 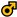 (UP Ar1051), Dush, Gjakovë, 42.53186° N, 20.59393° E, 500 m, riverside, collected by sweep net, 3 June 2025, leg: D.G., D.S.

**Distribution.** Europe, Turkey, Caucasus, Russia (Europe to Far East), Kazakhstan, Iran, Central Asia [1].

**Remarks.** The morphology of the male chelicera, especially the teeth, in conjunction with the shape and position of the embolus, allow recognition of this common species.

**Family Theridiidae Sundevall, 1833**

**Figure 34:**
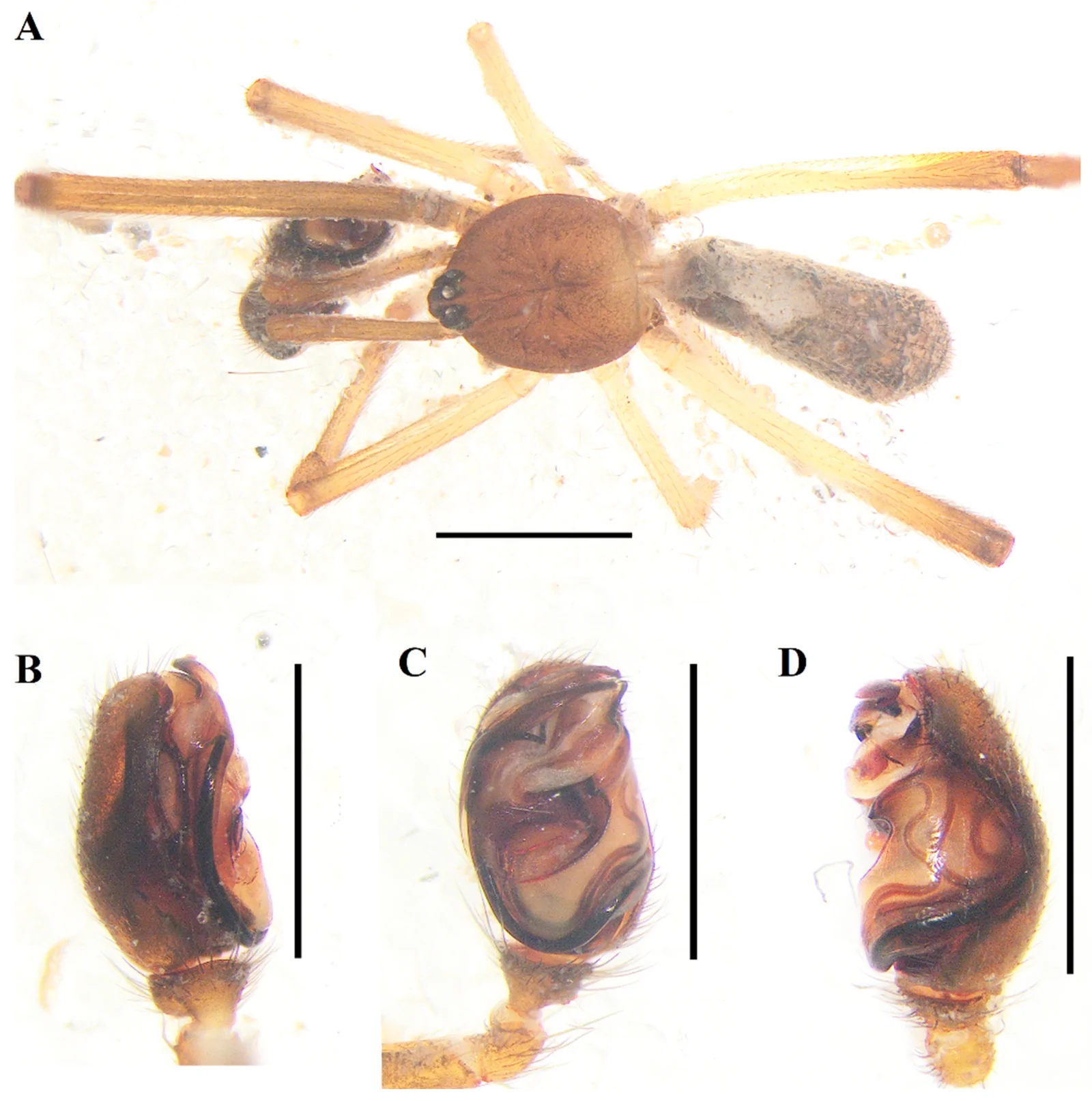
*Episinus truncatus* Latreille, 1809 male from Parku Germia, Kosovo: (**A**) habitus, dorsal view; (**B**) palp, prolateral view; (**C**) idem, ventral view; (**D**) idem, retrolateral view. Scale bars: 1 mm.

***Episinus truncatus* Latreille, 1809 (Figure 34)**

*Episinus truncatus*: Le Peru, 2011: 451, fig. 726 (mf) [28].

**Material examined.** 1 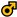 (UP Ar1052), Parku Germia, Prishtinë, 42.67566° N, 21.20271° E, 741 m, beech forest, vegetation beating, 25 April 2025, leg: D.G.

**Distribution.** Europe, Turkey, Caucasus, Iran [1].

**Remarks.** First record of the genus for the country. The course of the embolus and shape of the median apophysis allow recognition of the species.

**Figure 35:**
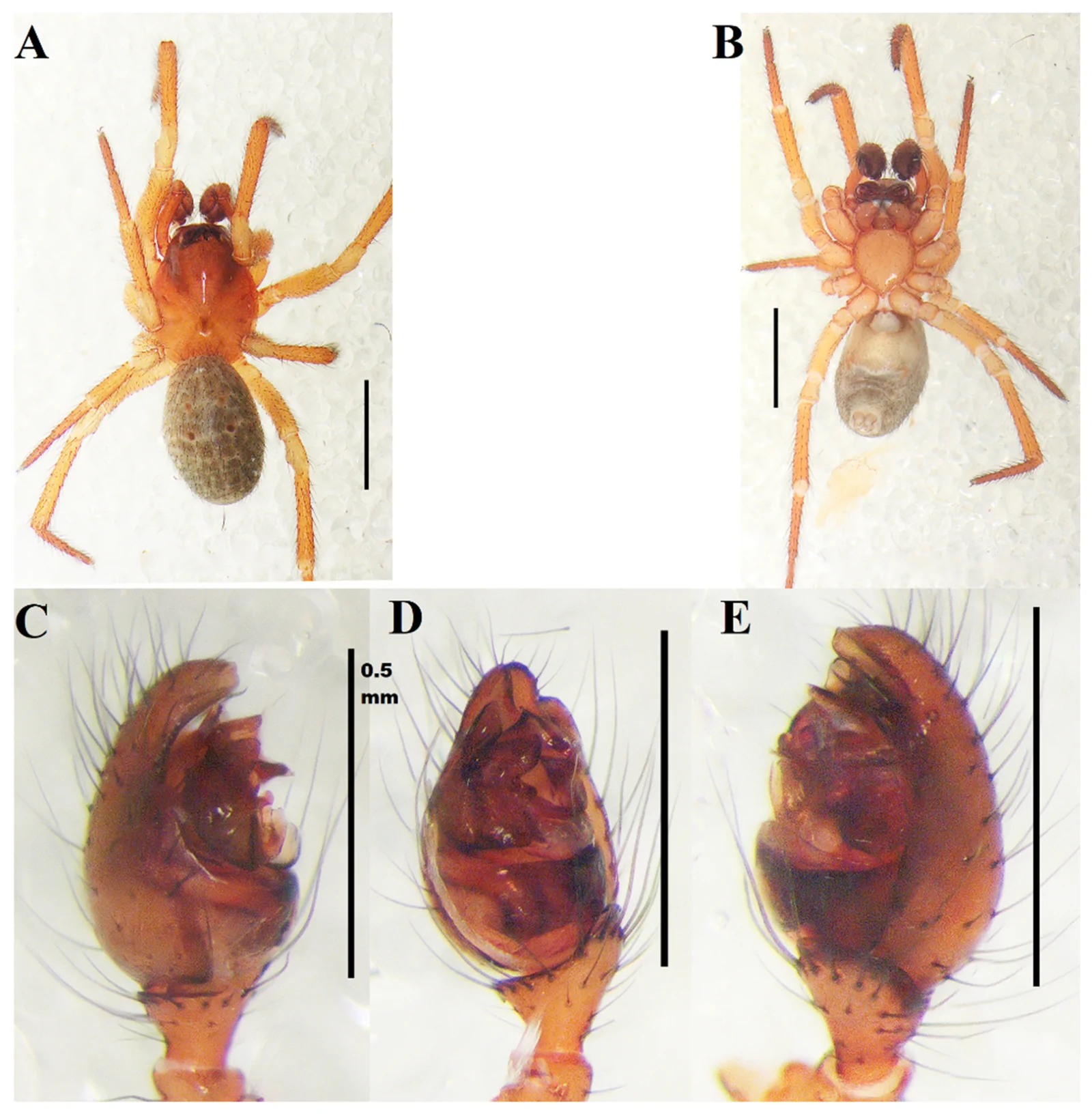
*Robertus mediterraneus* Eskov, 1987 male from Mokën, Kosovo: (**A**) habitus, dorsal view; (**B**) idem, ventral view; (**C**) palp, prolateral view; (**D**) idem, ventral view; (**E**) idem, retrolateral view. Scale bars: habitus (**A**,**B**) = 1 mm; palp (**C**–**E**) = 0.5 mm.

***Robertus mediterraneus* Eskov, 1987 (Figure 35)**

*Robertus mediterraneus*: Barrientos, Uribarri & García-Sarrión, 2014: 6, fig. 4 (mf) [40].

**Material examined.** 1 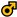 (UP Ar1053), Mokën, Zubin Potok, 42.90645° N, 20.55932° E, 1463 m, pine forest, collected by sieving leaflitter, 11 May 2026, leg: A.B., D.G., H.I.

**Distribution.** Spain, France, Switzerland, Austria, Italy, Slovenia, Eastern Europe, Caucasus [1].

**Remarks.** The shape of the embolic base and tip, together with the median apophysis, allow easy recognition of this species from other *Robertus* species in the Balkans.

**Figure 36:**
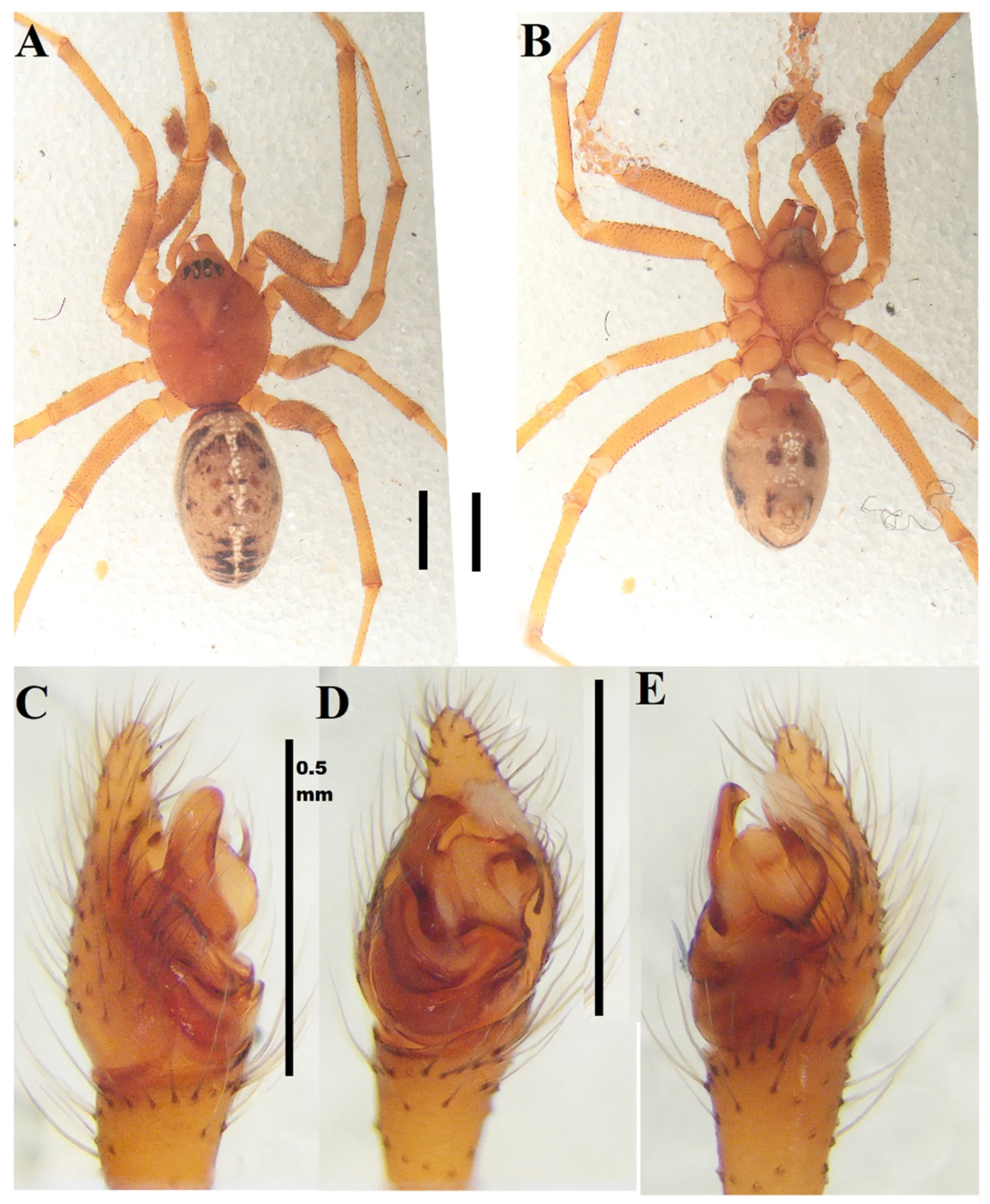
*Steatoda castanea* (Clerck, 1757) male from Veriq, Kosovo: (**A**) habitus, dorsal view; (**B**) idem, ventral view; (**C**) palp, prolateral view; (**D**) idem, ventral view; (**E**) idem, retrolateral view. Scale bars: habitus (**A**,**B**) = 1 mm; palp (**C**–**E**) = 0.5 mm.

***Steatoda castanea* (Clerck, 1757) (Figure 36)**

*Steatoda castanea*: Almquist, 2006: 92, figs. 114a–f (mf) [12].

**Material examined.** 2 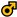 (UP Ar1054), Veriq, Istog, 42.75243° N, 20.55218° E, 510 m, inside house, collected by hand, 15 October 2025, leg: D.G.

**Distribution.** Europe, Turkey, Russia (Europe to Far East), Caucasus, Iran, Central Asia, China. Introduced to Canada [1].

**Remarks.** The shape of the embolus and its position behind the median apophysis and in front of the conductor allow ready identification of this species.

**Family Thomisidae Sundevall, 1833**

**Figure 37:**
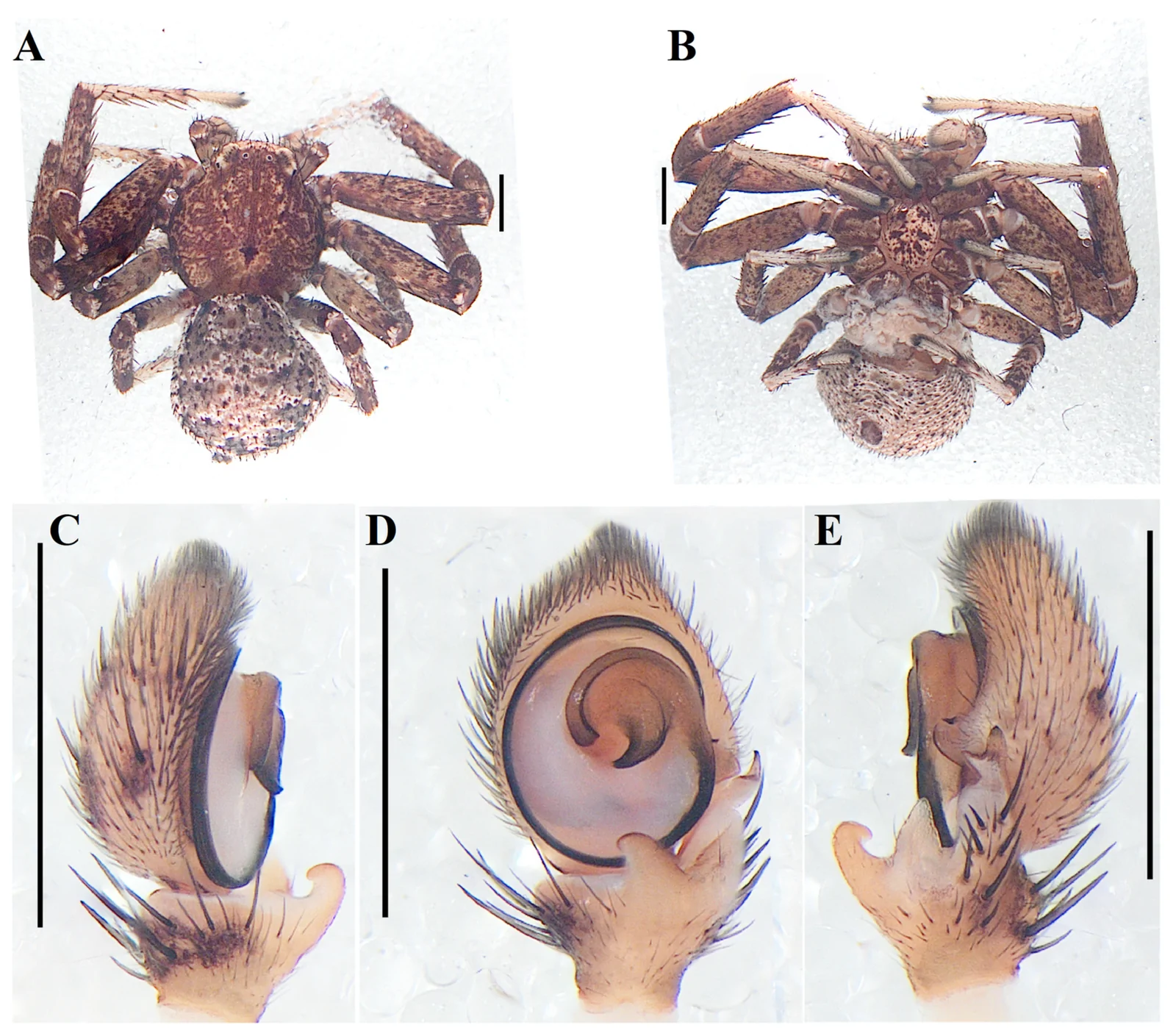
*Bassaniodes graecus* (C. L. Koch, 1837) male from Krevenik, Kosovo: (**A**) habitus, dorsal view; (**B**) idem, ventral view; (**C**) palp, prolateral view; (**D**) idem, ventral view; (**E**) idem, retrolateral view. Scale bars: 1 mm.

***Bassaniodes graecus* (C. L. Koch, 1837) (Figure 37)**

*Bassaniodes graecus*: Hernández-Corral, Barrientos & García-Teba, 2025: 61, figs. 13A-B (m) [41].

**Material examined.** 1 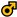 (UP Ar1055), Krevenik, Hani i Elezit, (site 2) 42.12160° N, 21.25456° E, 712 m, rocky escarpment, collected by hand, 30 May 2025, leg: D.G., D.S.

**Distribution.** Spain, Italy, Balkans, Greece, Ukraine, Russia (Europe), Turkey, Israel, Iraq [1].

**Remarks.** First record of the genus for the country. The curved median apophysis and spiralled embolus allow easy identification, combined with the shape of the palpal tibial apophyses.

**Figure 38:**
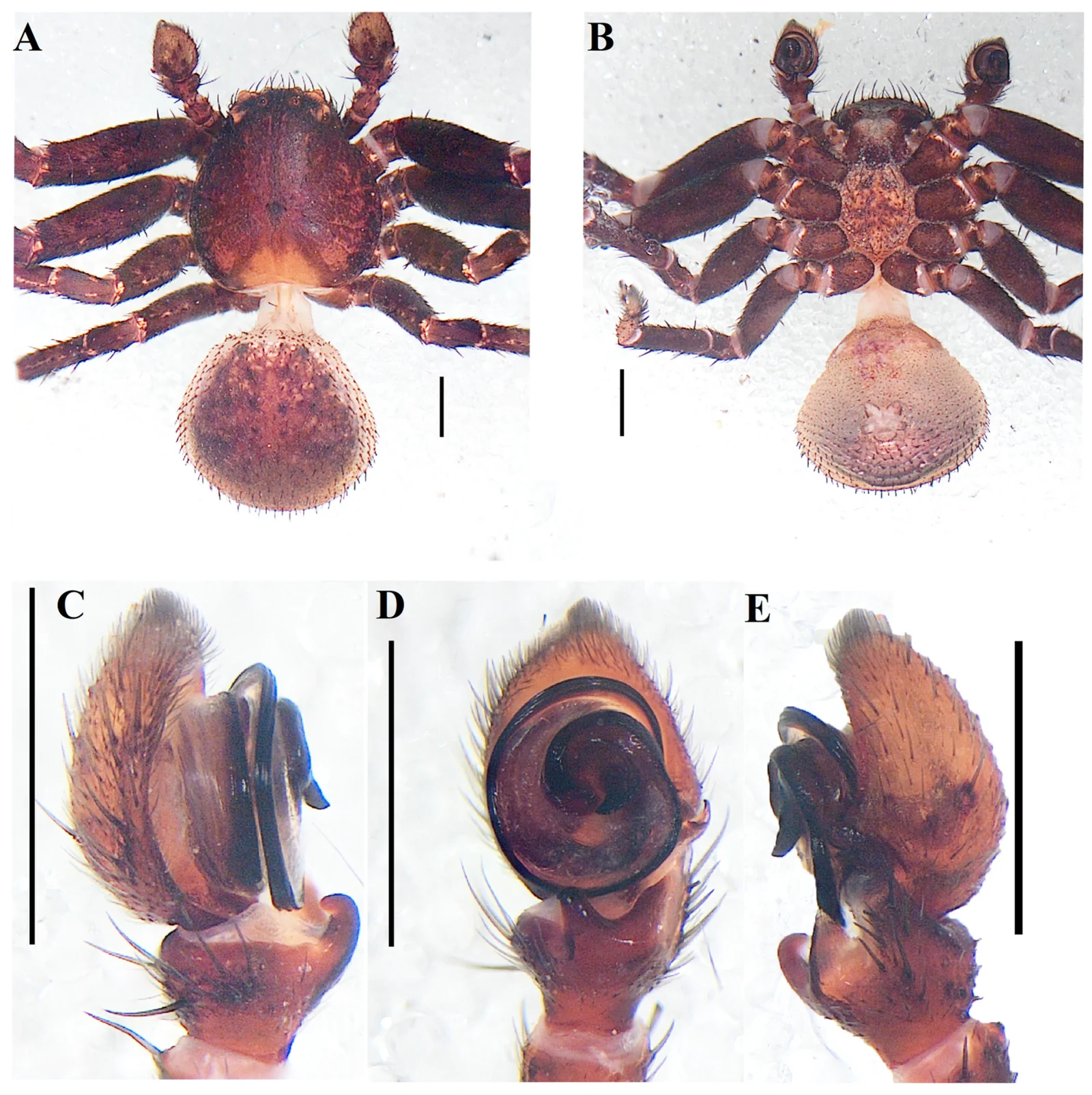
*Bassaniodes tenebrosus* (Šilhavý, 1944) male from Veriq, Kosovo: (**A**) habitus, dorsal view; (**B**) idem, ventral view; (**C**) palp, prolateral view; (**D**) idem, ventral view; (**E**) idem, retrolateral view. Scale bars: 1 mm.

***Bassaniodes tenebrosus* (Šilhavý, 1944) (Figure 38)**

*Xysticus tenebrosus*: Deltshev, Lazarov & Blagoev, 2004: 194, figs. 8–11 (mf) [42].

**Material examined.** 1 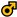 (UP Ar1056), Veriq, Istog, 42.75070° N, 20.55812° E, 543 m, oak forest, pitfall trap, 20 May 2025, leg: D.G.

**Distribution.** Slovenia to Turkey [1].

**Remarks.** The blunted median apophysis and thicker embolus allow differentiation from the *B. graecus* material examined herein.

**Figure 39:**
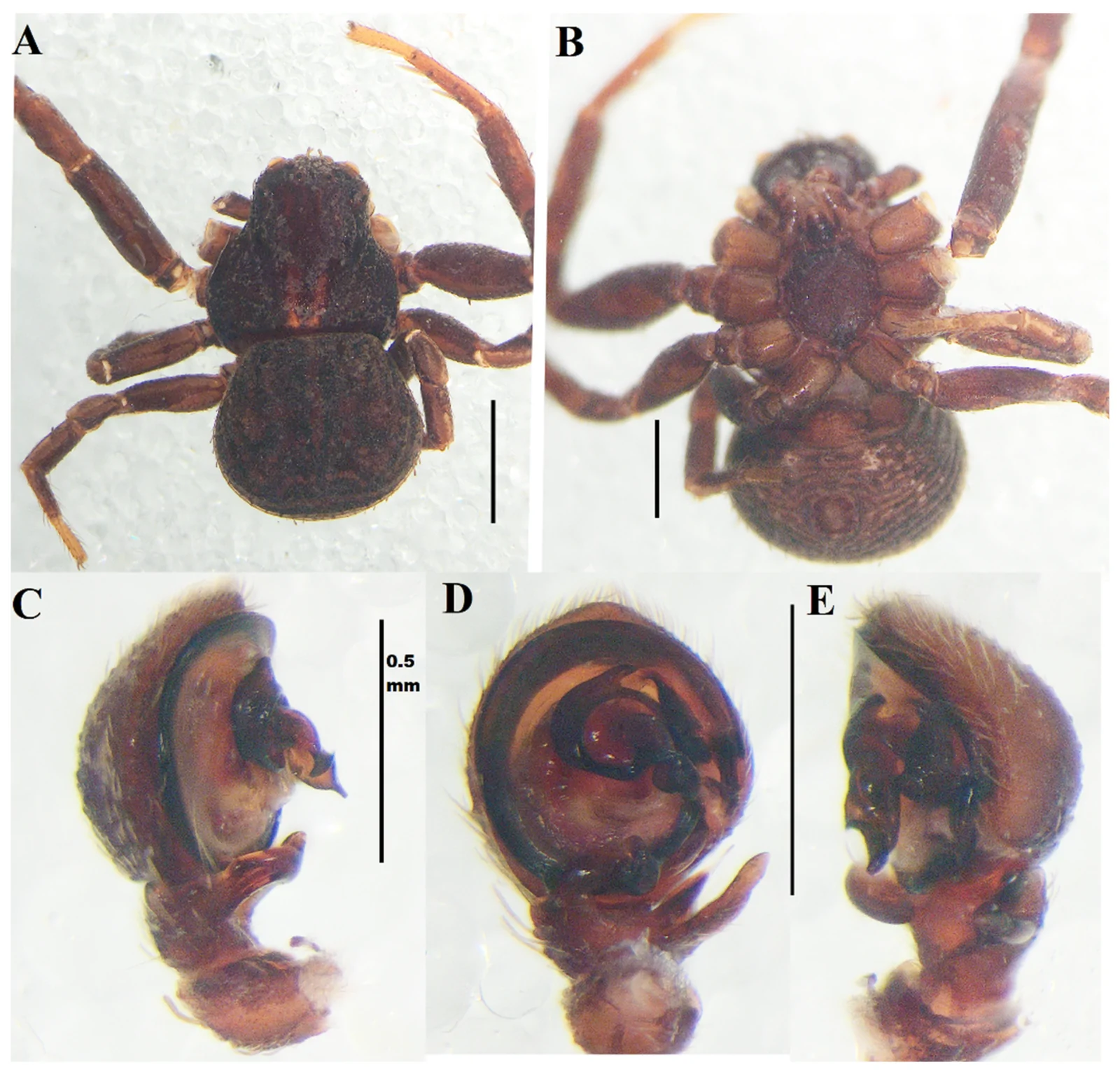
*Ozyptila pullata* (Thorell, 1875) male from Panorcë, Kosovo: (**A**) habitus, dorsal view; (**B**) idem, ventral view; (**C**) palp, prolateral view; (**D**) idem, ventral view; (**E**) idem, retrolateral view. Scale bars: habitus (**A**,**B**) = 1 mm; palp (**C**–**E**) = 0.5 mm.

***Ozyptila pullata* (Thorell, 1875) (Figure 39)**

*Ozyptila pullata*: Roberts, 1998: 176, unnumbered figs (mf) [43].

**Material examined.** 1 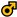 (UP Ar1057), Panorcë, Malishevë, 42.48824° N, 20.62089° E, 571 m, oak forest, collected by sieving leaflitter, 15 July 2020, leg: A.B., D.G., M.M.

Distribution. Europe [1].

**Remarks.** The structure of the embolus, median apophysis, and retrolateral tibial apophysis allow recognition of this species.

**Family Zodariidae Thorell, 1881**

**Figure 40:**
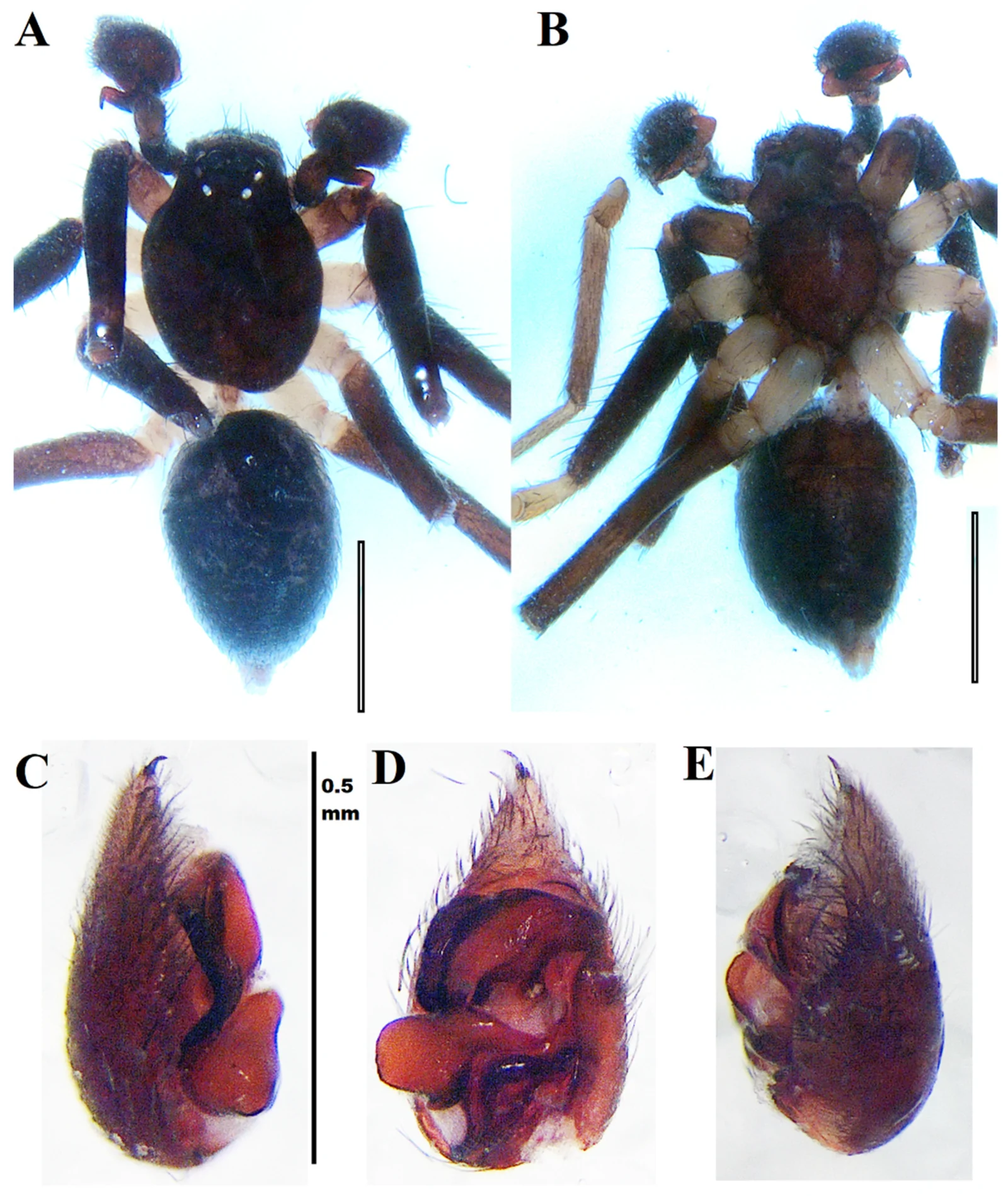
*Zodarion morosum Denis*, 1935 male from Krevenik, Kosovo: (**A**) habitus, dorsal view; (**B**) idem, ventral view; (**C**) cymbium (dissected), prolateral view; (**D**) idem, ventral view; (**E**) idem, retrolateral. Scale bars: habitus (**A**,**B**) = 1 mm; palp (**C**–**E**) = 0.5 mm.

***Zodarion morosum* Denis, 1935 (Figure 40)**

*Zodarion morosum*: Shafaie, Pekár & Seyyar, 2025: 100, figs. 78A–D, 79A-E (mf) [44].

**Material examined.** 1 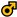 (UP Ar1058), Krevenik, Hani i Elezit, (site 2) 42.12160° N, 21.25456° E, 712 m, rocky escarpment, found near ant nest, collected by hand, 30 May 2025, leg: D.G., D.S.

**Distribution.** Albania, North Macedonia, Bulgaria, Greece, Romania, Ukraine, Russia (Europe, Caucasus), Georgia, Turkey, Syria [1].

**Remarks.** The wide projection on the tegulum and inconspicuously positioned embolus allow differentiation from other congeners.

## 4. Discussion

This study significantly increases current knowledge of the spider fauna of Kosovo by documenting numerous species, genera, and families recorded for the first time in the country. Considering the relatively limited sampling effort and geographic coverage of the present work, the large number of new national records strongly suggests that the actual spider diversity of Kosovo is substantially underestimated.

The newly recorded taxa belong to a wide range of ecological and biogeographical groups, including widespread European species, Mediterranean elements, and taxa associated with montane and forest habitats. Several species recorded in this study, (e.g., *Oxyopes nigripalpis*, *Bassaniodes tenebrosus*, and *Harpactea coccifera*), are characteristic of southern European and Mediterranean regions, reflecting the transitional biogeographic position of Kosovo between continental and Mediterranean climatic influences [45]. In contrast, other species (e.g., *Acantholycosa lignaria*, *Bathyphantes nigrinus*, and *Pocadicnemis juncea*) are more typical of cooler montane or temperate habitats, emphasizing the ecological heterogeneity of the country.

The discovery of two spider families previously unrecorded from Kosovo, namely Cicurinidae and Oonopidae, is particularly important. These findings demonstrate that even higher taxonomic groups remain undocumented in the country despite increasing arachnological research in recent years. This highlights the importance of targeted sampling methods such as litter sifting and microhabitat-focused collecting for detecting cryptic taxa that are easily overlooked during general surveys.

Many of the newly recorded species are widely distributed across Europe and the western Palearctic (e.g., *Larinioides cornutus*, *Dictyna arundinacea*, *Tetragnatha montana*, and *Steatoda castanea*). Their absence from previous records in Kosovo likely reflects insufficient historical sampling rather than genuine rarity. Similar patterns have been documented in neighbouring Balkan countries, where recent surveys have substantially increased the known number of spider taxa [2,8,31].

Our work increases the known the number spider fauna of Kosovo to a total of 322 species, 160 genera, and 37 families. The numerous new country records reported here emphasise the urgent need for continued arachnological exploration in Kosovo and neighbouring countries. Future taxonomic, ecological, and molecular investigations will undoubtedly continue to expand our understanding of the distribution, diversity, and biogeographic significance of the Kosovar arachnofauna.

## Data Availability

All data is available in the present paper.

## References

[1] World Spider Catalog 2026. World Spider Catalog, Version 27. Natural History Museum Bern. http://wsc.nmbe.ch.

[2] Naumova M., Lazarov S., Deltshev C. (2019). Faunistic diversity of the spiders in Montenegro (Arachnida: Araneae). Ecol. Montenegrina.

[3] Geci D., Naumova M. (2021). A preliminary checklist of the spiders of Kosovo (Arachnida: Araneae). Ecol. Balk..

[4] Geci D., Naumova M., Ibrahimi H., Grapci-Kotori L., Gashi A., Bilalli A., Musliu M. (2023). Contribution to spider fauna (Arachnida: Araneae) from Bjeshkët e Nemuna mountains (Kosovo). Nat. Croat..

[5] Geci D., Ibrahimi H., Naumova M., Bilalli A., Koblmüller S., Musliu M., Grapci-Kotori L., Gashi A., Schäffer S., Wagner M. (2025). Description of a new *Eratigena* Bolzern, Burckhardt & Hänggi, 2013 (Araneae: Agelenidae) from the Balkans based on an integrative approach. Zootaxa.

[6] Geci D., Ibrahimi H., Strohmeier T., Bilalli A., Musliu M., Koblmüller S., Sherwood D. (2025). Trapdoor spiders of the family Nemesiidae Simon, 1889 (Araneae: Mygalomorphae) from Kosovo. Arachnology.

[7] Geci D., Ibrahimi H., Strohmeier T., Koblmüller S., Bilalli A., Zamani A. (2025). First DNA barcoding of Dysderidae (Araneae) from Kosovo, with new records and the description of a new species of *Harpactea* Bristowe, 1939. Zootaxa.

[8] Geci D., Ibrahimi H., Bilalli A., Musliu M. (2025). New records of spiders (Arachnida: Araneae) from the Western Balkans. J. Insect Biodivers. Syst..

[9] Nentwig W., Blick T., Bosmans R., Kropf C., Stäubli A. Spiders of Europe. Version 05.2026. https://www.araneae.nmbe.ch.

[10] Lecigne S. (2021). A new species of *Sintula* (Linyphiidae), redescription of *Brigittea innocens* (Dictynidae) and eight spider species newly recorded for Turkey (Araneae). Arachnol. Mitt..

[11] Marusik Y.M., Böcher J., Kristensen N.P., Pape T., Vilhelmsen L. (2015). Araneae (Spiders). The Greenland Entomofauna. An Identification Manual of Insects, Spiders and Their Allies.

[12] Almquist S. (2006). Swedish Araneae, Part 1—Families Atypidae to Hahniidae (Linyphiidae excluded).

[13] Esyunin S.L. (2017). A D.E. Kharitonov’s collection of permanent slides of spiders (Arachnida, Araneae). Part 1. Family Dictynidae. Bull. Perm Univ. Biol..

[14] Brignoli P.M. (1984). Ragni di Grecia XII. Nuovi dati su varie famiglie (Araneae). Rev. Suisse Zool..

[15] Almquist S. (2007). Swedish Araneae, part 2—Families Dictynidae to Salticidae. Insect Systematics & Evolution.

[16] Indzhov S., Haynadzhieva V., Střeštík V., Dimitrov D., Dimitrov D. (2025). We have two of them: A review of the genus *Mastigusa* (Araneae: Cybaeidae) in Bulgaria. Zootaxa.

[17] Ponomarev A.V., Shmatko V.Y. (2022). A new species and new records of spiders (Aranei) in the south of European Russia. Cauc. Entomol. Bull..

[18] Barrientos J.A., Moya J., García-Sarrión R., Uribarri I., Melic A., Prieto C.E., Moraza M.L., Zaragoza J.A. (2017). Arácnidos del Parque Natural de Cabo de Gata-Níjar (Almería, España). Rev. Ibér. Aracnol..

[19] Lecigne S. (2016). Contribution à la connaissance de l’aranéofaune (Araneae) de Crète (Grèce) et description de la femelle inconnue de *Neaetha absheronica* Logunov & Guseinov, 2002 (Salticidae). Nieuwsbr. Belg. Arachnol. Ver..

[20] Ponomarev A.V., Shmatko V.Y. (2020). A review of spiders of the genera *Trachyzeloes* [*sic*] Lohmander, 1944 and *Marinarozelotes* Ponomarev, gen. n. (Aranei: Gnaphosidae) from the southeast of the Russian Plain and the Caucasus. Cauc. Entomol. Bull..

[21] Lecigne S. (2020). Sur quelques observations intéressantes d’araignées du Nord et du Pas-de-Calais (France)—1re note. Le Héron.

[22] Bosselaers J. (2018). Spiders (Arachnida, Araneae) of the Gavarres (Catalonia, Spain) and the adjacent coastal region—Part I: 2012–2013. Newsl. Belg. Arachnol. Soc..

[23] Otto S., Japoshvili G. (2018). The spiders (Arachnida: Araneae) of the Lagodekhi Reserve, Georgia: Faunistic results of a transect study and an updated checklist. Arachnology.

[24] Danışman T. (2014). First record of the linyphiid spider *Walckenaeria furcillata* (Menge, 1869) (Araneae, Linyphiidae) in Turkey. Serket.

[25] Marusik Y.M., Omelko M.M. (2011). A survey of East Palaearctic Lycosidae (Araneae). 7. A new species of *Acantholycosa* Dahl, 1908 from the Russian Far East. ZooKeys.

[26] Bayram A., Efil L., Deltshev C. (2009). *Pardosa roscai* (Roewer, 1951), a spider new to the fauna of Turkey (Araneae: Lycosidae). Turk. J. Zool..

[27] Thaler K. (1987). *Pardosa vittata* (Keyserling)—Neu für Österreich—Und weitere Wolfspinnen aus dem Kulturland des Grazer Beckens (Araneae, Lycosidae). Sitzungsber. Osterr. Akad. Wiss..

[28] Le Peru B. (2011). The spiders of Europe, a synthesis of data: Volume 1 Atypidae to Theridiidae. Mém. Société Linn. Lyon.

[29] Esyunin S.L., Tuneva T.K. (2009). A review of Palaearctic lynx-spiders of the *heterophthalmus* group of the genus *Oxyopes* (Aranei, Oxyopidae). Zool. Zhurnal.

[30] Tchilinguirian J., Sherwood D. (2025). The occasionally broken tibial apophysis of male *Oxyopes heterophthalmus* (Latreille, 1804) (Araneae: Oxyopidae). Rev. Ibér. Aracnol..

[31] Naumova M., Blagoev G., Deltshev C. (2021). Fifty spider species new to the Bulgarian fauna, with a review of some dubious species (Arachnida: Araneae). Zootaxa.

[32] Kastrygina Z.A., Kovblyuk M.M. (2016). Vicariance of two closely related spider species from genus *Philodromus* Walckenaer, 1826: *P. albidus* Kulczynski, 1911 and *P. rufus* Walckenaer, 1826 (Aranei, Philodromidae) in the Crimea. Sci. Notes Crime. Fed. VI Vernadsky Univ. Ser. Biol. Chem..

[33] Roberts M.J. (1993). Appendix to: The Spiders of Great Britain and Ireland.

[34] Marta K.S., Bustamante A.A., Hagopián D., Teixeira R.A., Brescovit A.D., Valiati V.H., Rodrigues E.N.L. (2024). Taxonomic revision of the jumping spider genus *Tullgrenella* Mello-Leitão, 1941 (Araneae: Salticidae: Freyina). Zootaxa.

[35] Metzner H. (1999). Die Springspinnen (Araneae, Salticidae) Griechenlands. Andrias.

[36] Logunov D.V., Schäfer M. (2025). A review of the jumping spider genus *Macaroeris* Wunderlich, 1992 (Arachnida: Araneae: Salticidae: Dendryphantini). Arachnology.

[37] Schäfer M. (2021). Ein Beitrag zur Springspinnenfauna (Araneae: Salticidae) der griechischen Insel Korfu mit vier Erstnachweisen für die Insel und Anmerkungen zur Gattung Salticus. Arachnol. Mitt..

[38] Seropian A., Bulbulashvili N., Otto S., Krammer H.-J., Kachlishvili N., Datunashvili A. (2023). Picking pearls from the Silk Road: Insights into the spider (Arachnida, Araneae) diversity in Georgia from the Caucasus barcode of life project. Part II. Caucasiana.

[39] Morano E. (2020). La familia Tetragnathidae Menge, 1866 (Araneae) en el área ibero-balear. Rev. Ibér. Aracnol..

[40] Barrientos J.A., Uribarri I., García-Sarrión R. (2014). Arañas (Arachnida, Araneae) de un hayedo del Montseny (Cataluña, España). Rev. Ibér. Aracnol..

[41] Hernández-Corral J., Barrientos J.A., García-Teba J.P. (2025). Nuevos datos sobre las arañas (Arachnida: Araneae) del Parc Natural del Carrascal de la Font Roja (Alicante, España). Rev. Ibér. Aracnol..

[42] Deltshev C., Lazarov S., Blagoev G., Beron P., Popov A. (2004). Spiders (Araneae) from the eastern Rhodopes (Bulgaria and Greece). Biodiversity of Bulgaria, 2. Biodiversity of Eastern Rhodopes (Bulgaria and Greece).

[43] Roberts M.J. (1998). Spinnengids.

[44] Shafaie S., Pekár S., Seyyar O. (2025). New data on zodariid spiders (Arachnida, Araneae) in the Middle East and Eastern Mediterranean. Eur. J. Taxon..

[45] Berisha N., Krasniqi E., Bytyqi V. (2025). Biogeographical regionalization of Kosovo: Integrating vegetation, climate and topography. Mediterr. Bot..

